# Supplementary material for: Synthesis and Biological Activity of Unsymmetrical Monoterpenylhetaryl Disulfides
Source: Molecules. 2022 Aug 10;27(16):5101. doi: 10.3390/molecules27165101 (PMC9416111; doi:10.3390/molecules27165101)
Supplement: Supplementary file 1 [file molecules-27-05101-s001.zip › molecules-1852353-supplementary.pdf]

Molecules

Supplementary Materials

Synthesis and biological activity of unsymmetrical monoterpenylhetaryl disulfides

Denis V. Sudarikov*, Yulia V. Gyrdymova, Alexander V. Borisov, Julia M. Lukiyanova, Roman V. Rumyantcev, Oksana G. Shevchenko, Diana R. Baidamshina, Nargiza D Zakarova, Airat R. Kayumov, Ekaterina O. Sinegubova, Aleksandrina S. Volobueva, Vladimir V. Zarubaev, Svetlana A. Rubtsova

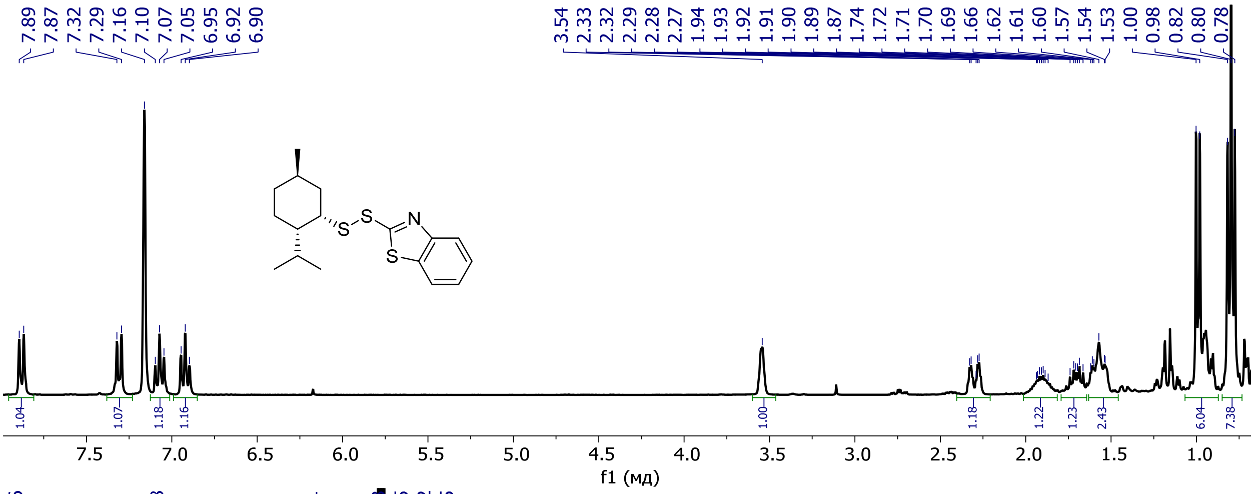
7.89
7.87
7.32
7.29
7.16
7.10
7.07
7.05
6.95
6.92
6.90
3.54
2.33
2.32
2.29
2.28
2.27
1.94
1.93
1.92
1.91
1.90
1.89
1.87
1.74
1.72
1.71
1.70
1.69
1.66
1.62
1.61
1.60
1.57
1.54
1.53
1.00
0.98
0.82
0.80
0.78
1.04
1.07
1.18
1.16
1.00
1.18
1.22
1.23
2.43
6.04
7.38
f1 (мд)

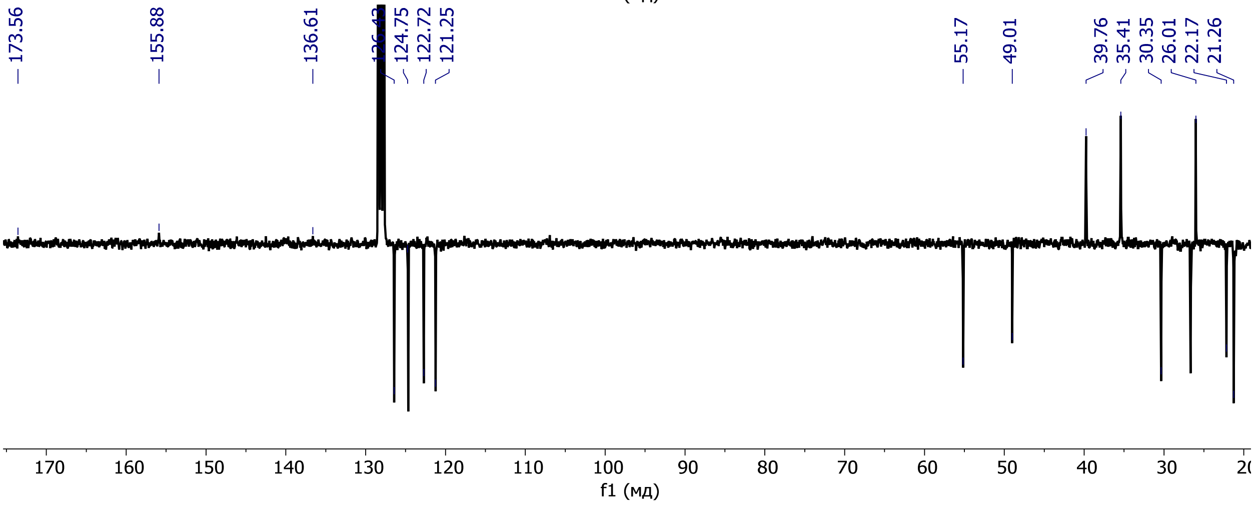
173.56
155.88
136.61
124.75
122.72
121.25
55.17
49.01
39.76
35.41
30.35
26.01
22.17
21.26
f1 (мд)

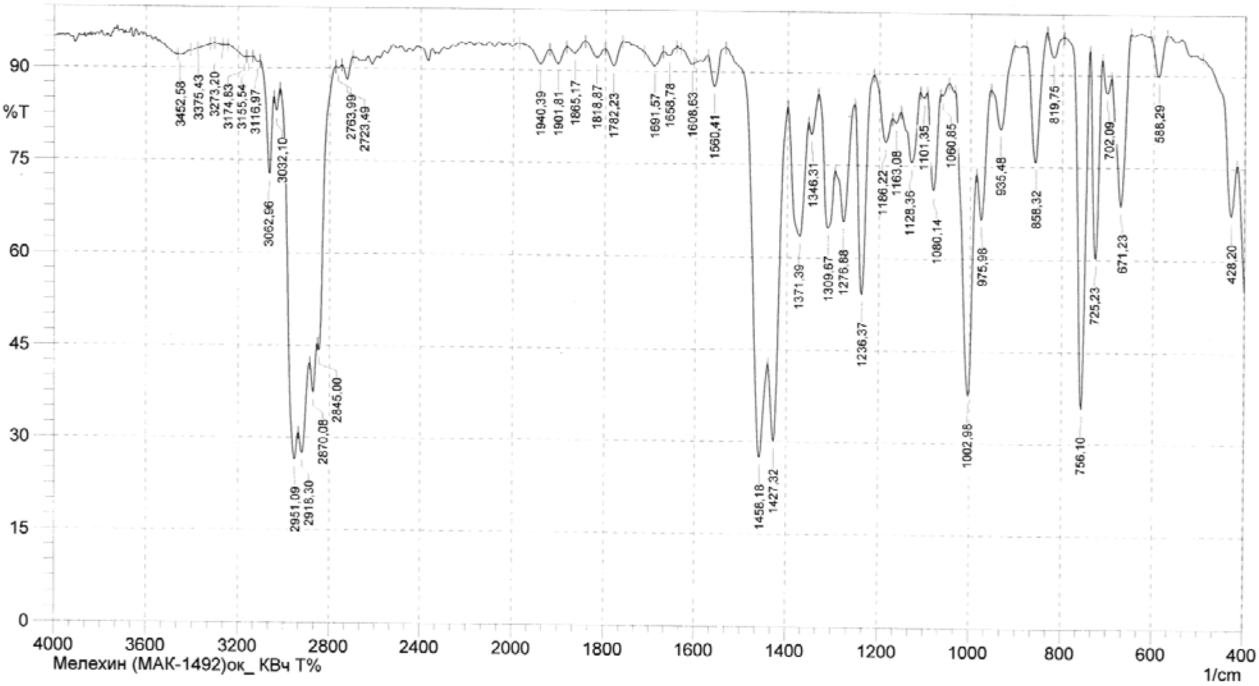
%T
1/cm
Мелехин (МАК-1492)ок_ КВч Т%

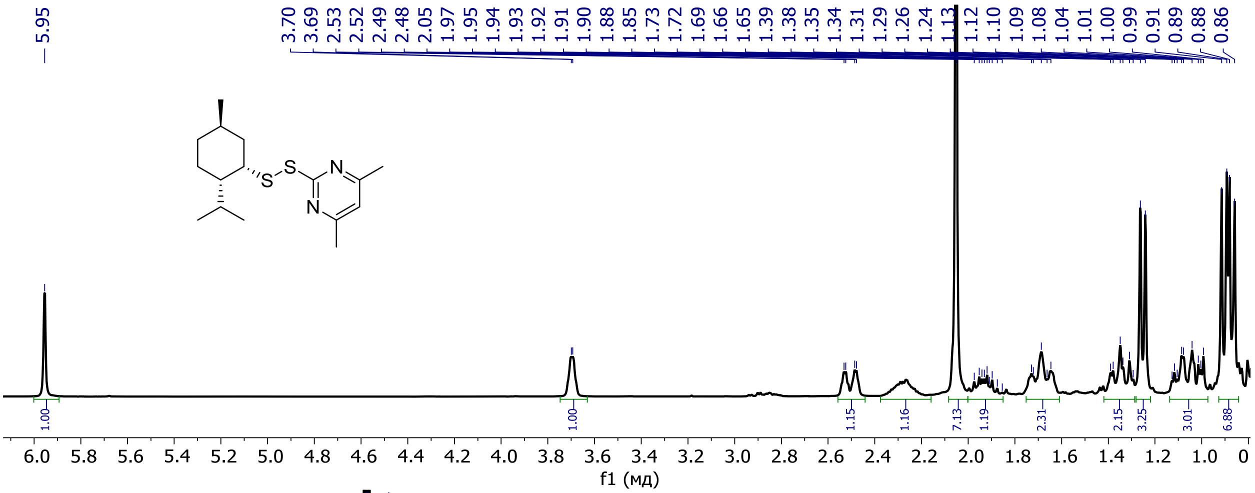
5.95
3.70 3.69 2.53 2.52 2.49 2.48 2.05 1.97 1.95 1.94 1.93 1.92 1.91 1.90 1.88 1.85 1.73 1.72 1.69 1.66 1.65 1.39 1.38 1.35 1.34 1.31 1.29 1.26 1.24 1.13 1.12 1.10 1.09 1.08 1.04 1.01 1.00 0.99 0.91 0.89 0.88 0.86
S
S
N
N
1.00 1.00 1.15 1.16 7.13 1.19 2.31 2.15 3.25 3.01 6.88
6.0 5.8 5.6 5.4 5.2 5.0 4.8 4.6 4.4 4.2 4.0 3.8 3.6 3.4 3.2 3.0 2.8 2.6 2.4 2.2 2.0 1.8 1.6 1.4 1.2 1.0 0
f1 (мд)

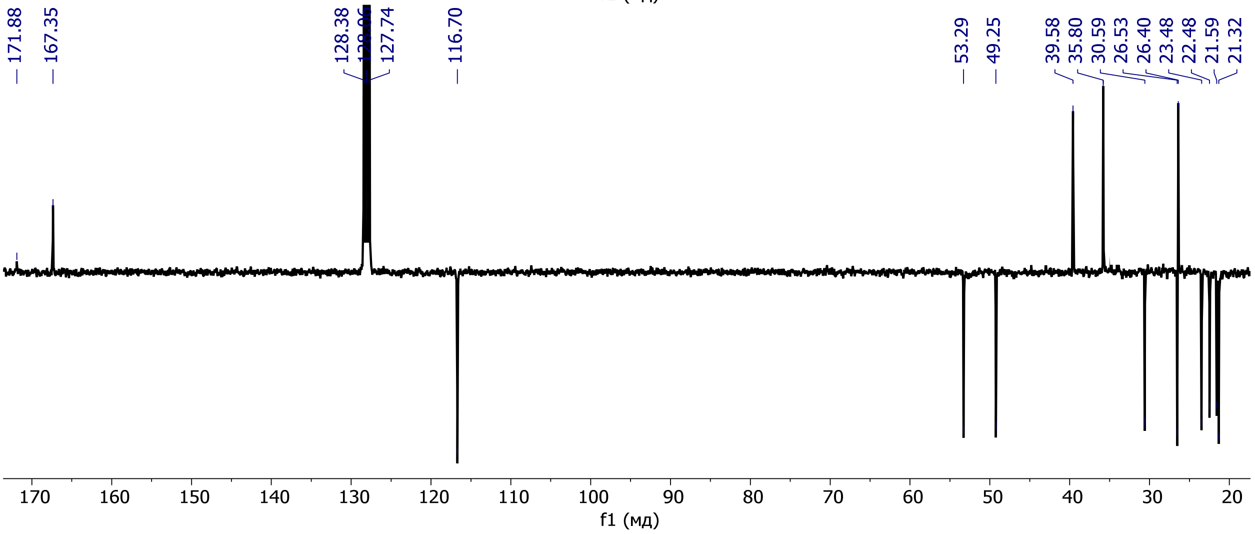
171.88
167.35
128.38
127.74
116.70
53.29
49.25
39.58 35.80 30.59 26.53 26.40 23.48 22.48 21.59 21.32
170 160 150 140 130 120 110 100 90 80 70 60 50 40 30 20
f1 (мд)

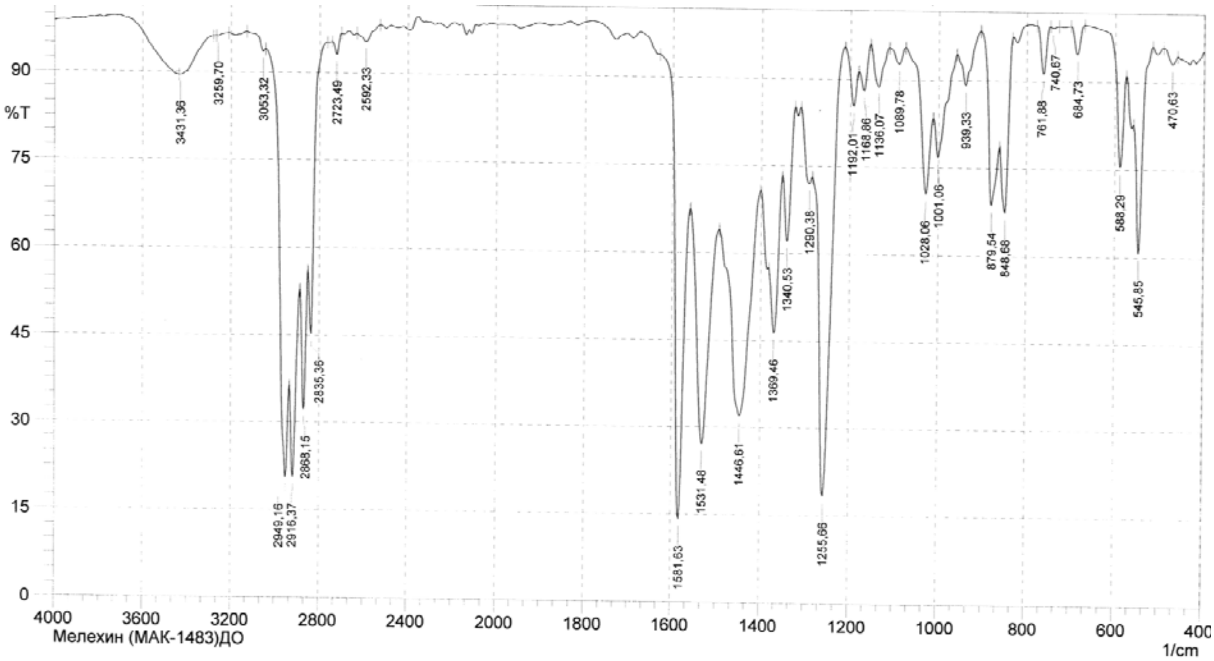
%T
90 75 60 45 30 15 0
3431,36 3259,70 3053,32 2723,49 2592,33 2949,16 2916,37 2868,15 2835,36
1581,63 1531,48 1446,61 1369,46 1340,53 1290,38 1255,66 1192,01 1168,86 1136,07 1089,78 1028,06 1001,06 939,33 879,54 848,68 761,88 740,67 684,73 588,29 545,85 470,63
4000 3600 3200 2800 2400 2000 1800 1600 1400 1200 1000 800 600 400
1/cm
Мелехин (МАК-1483)ДО

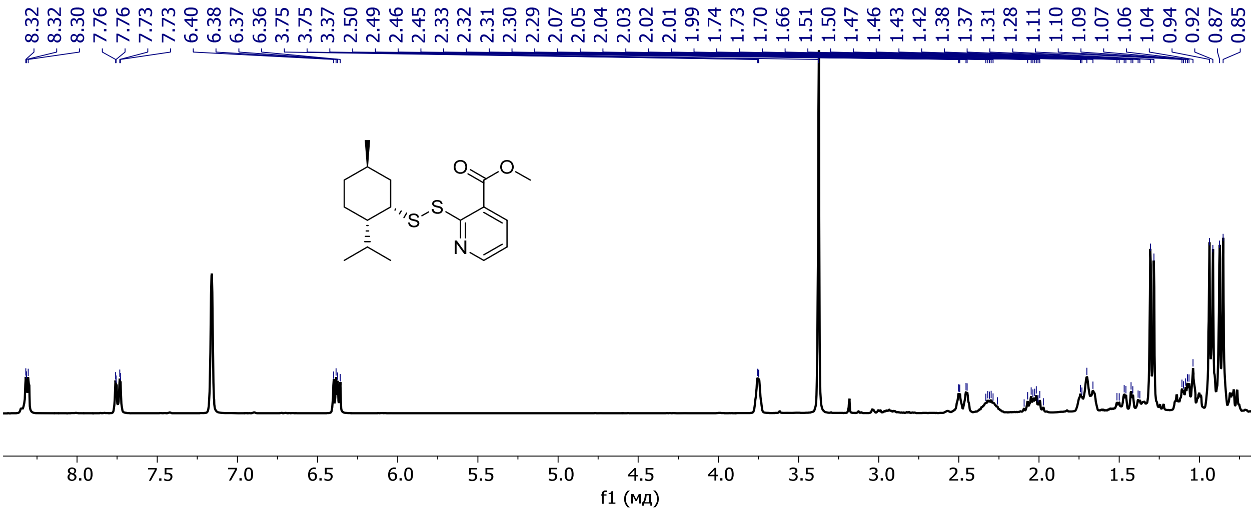

8.32 8.32 8.30 7.76 7.76 7.73 7.73 6.40 6.38 6.37 6.36 3.75 3.75 3.37 2.50 2.49 2.46 2.45 2.33 2.32 2.31 2.30 2.29 2.07 2.05 2.04 2.03 2.02 2.01 1.99 1.74 1.73 1.70 1.66 1.51 1.50 1.47 1.46 1.43 1.42 1.38 1.37 1.31 1.28 1.11 1.10 1.09 1.07 1.06 1.04 0.94 0.92 0.87 0.85
O
O
S
S
N
8.0 7.5 7.0 6.5 6.0 5.5 5.0 4.5 4.0 3.5 3.0 2.5 2.0 1.5 1.0
f1 (мд)

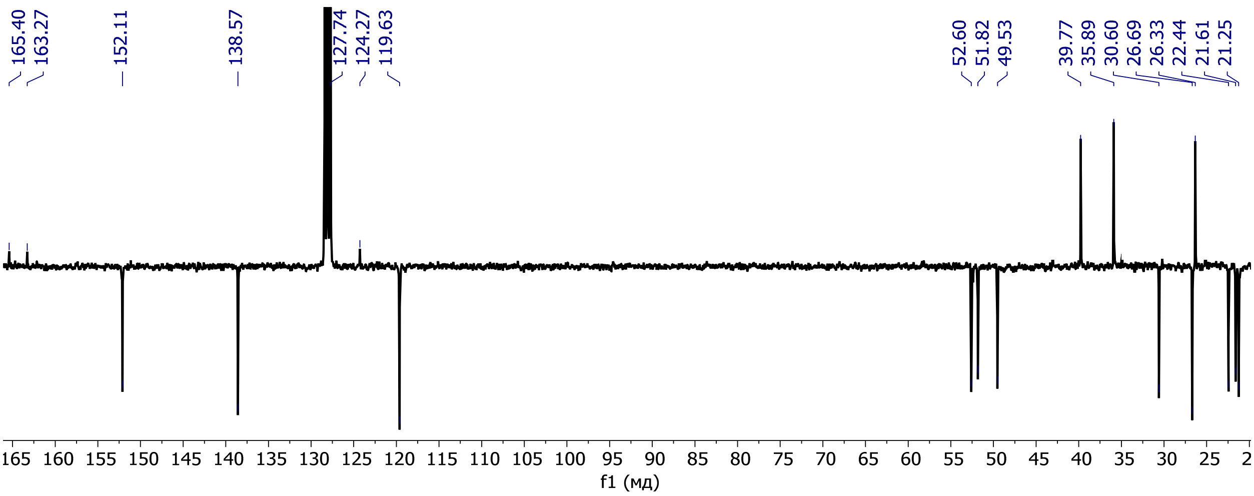

165.40 163.27 152.11 138.57 127.74 124.27 119.63 52.60 51.82 49.53 39.77 35.89 30.60 26.69 26.33 22.44 21.61 21.25
165 160 155 150 145 140 135 130 125 120 115 110 105 100 95 90 85 80 75 70 65 60 55 50 45 40 35 30 25 2
f1 (мд)

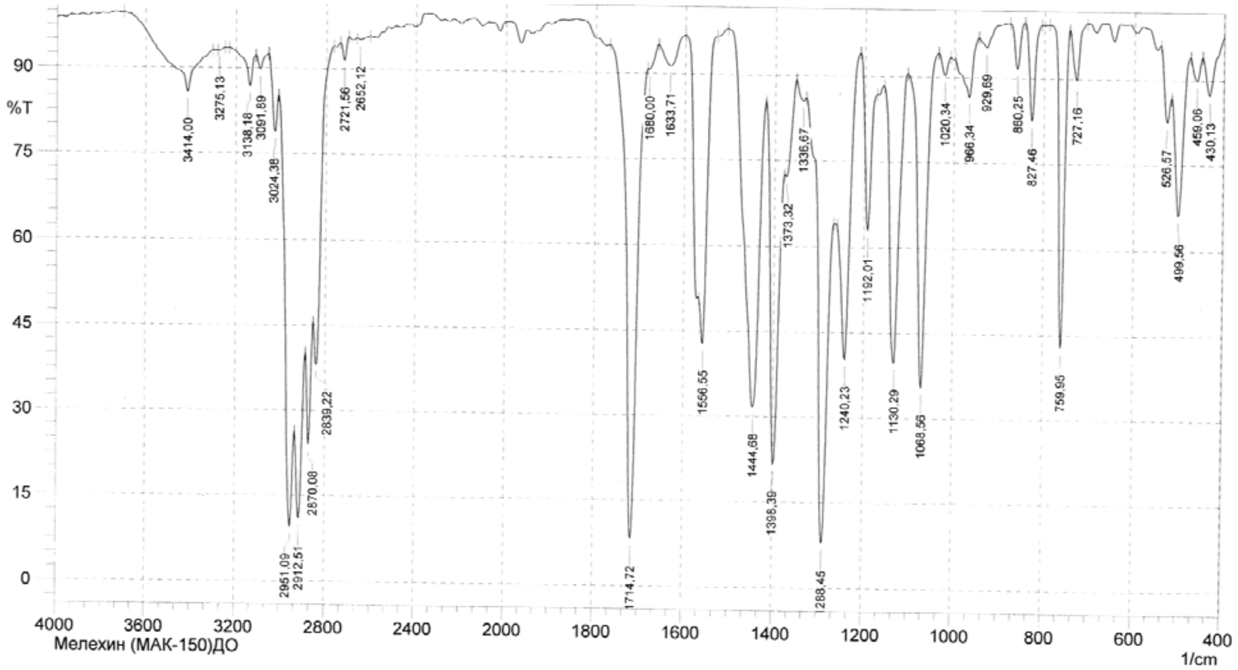

90
%T
75
60
45
30
15
0
3414,00 3275,13 3138,18 3091,89 3024,38 2951,09 2912,51 2870,08 2839,22 2721,56 2652,12 1714,72 1680,00 1633,71 1556,55 1444,68 1398,39 1373,32 1336,67 1288,45 1240,23 1192,01 1130,29 1068,56 1020,34 966,34 929,69 860,25 827,46 759,95 727,16 526,57 499,56 459,06 430,13
4000 3600 3200 2800 2400 2000 1800 1600 1400 1200 1000 800 600 400
Мелехин (МАК-150)ДО
1/cm

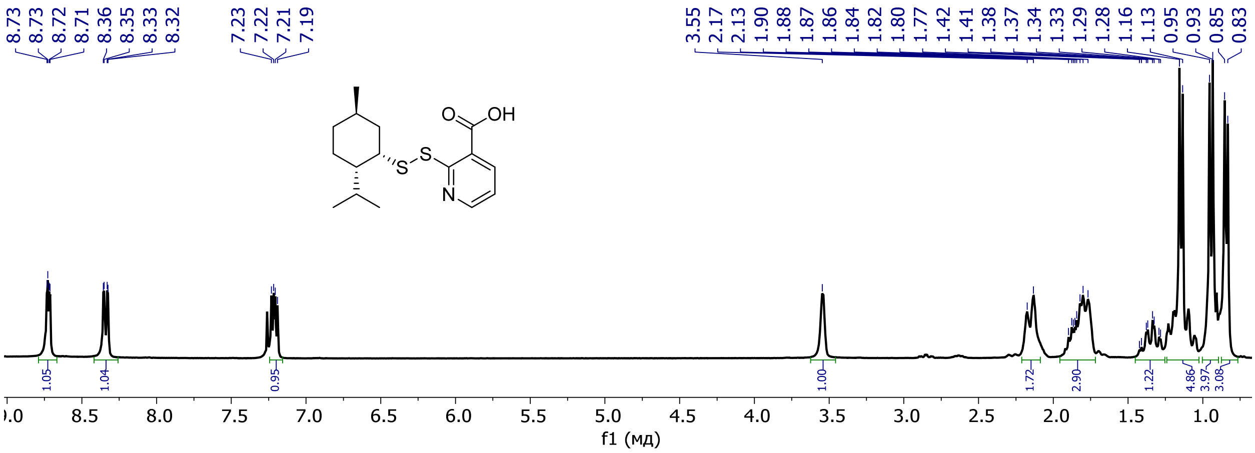
8.73 8.73 8.72 8.71 8.36 8.35 8.33 8.32
7.23 7.22 7.21 7.19
3.55 2.17 2.13 1.90 1.88 1.87 1.86 1.84 1.82 1.80 1.77 1.42 1.41 1.38 1.37 1.34 1.33 1.29 1.28 1.16 1.13 0.95 0.93 0.85 0.83
O OH
S S
N
1.05 1.04 0.95 1.00 1.72 2.90 1.22 4.86 3.97 3.08
f1 (мд)

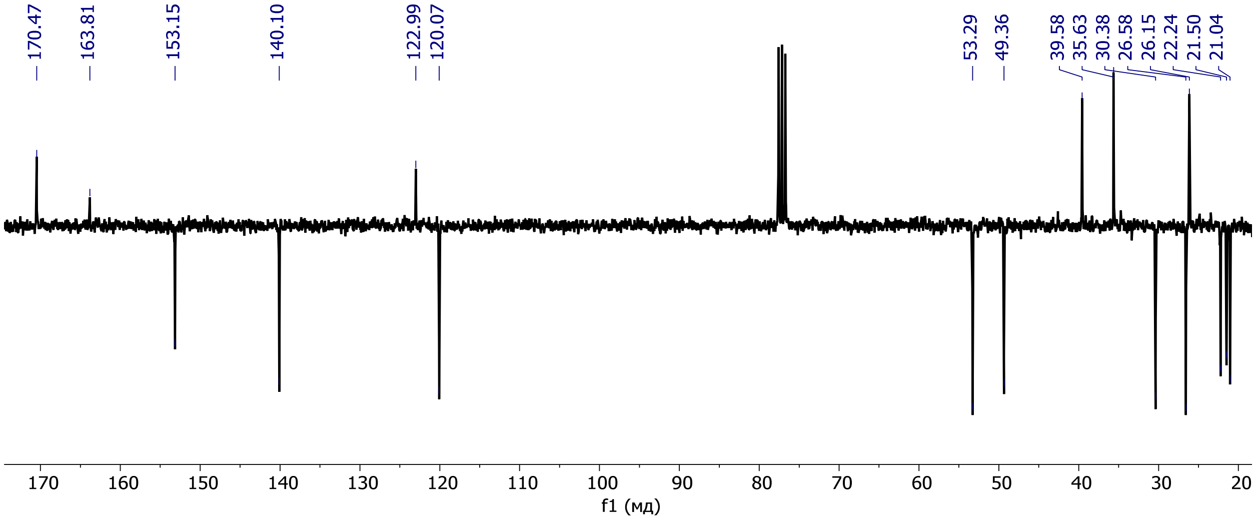
170.47 163.81 153.15 140.10 122.99 120.07 53.29 49.36 39.58 35.63 30.38 26.58 26.15 22.24 21.50 21.04
f1 (мд)

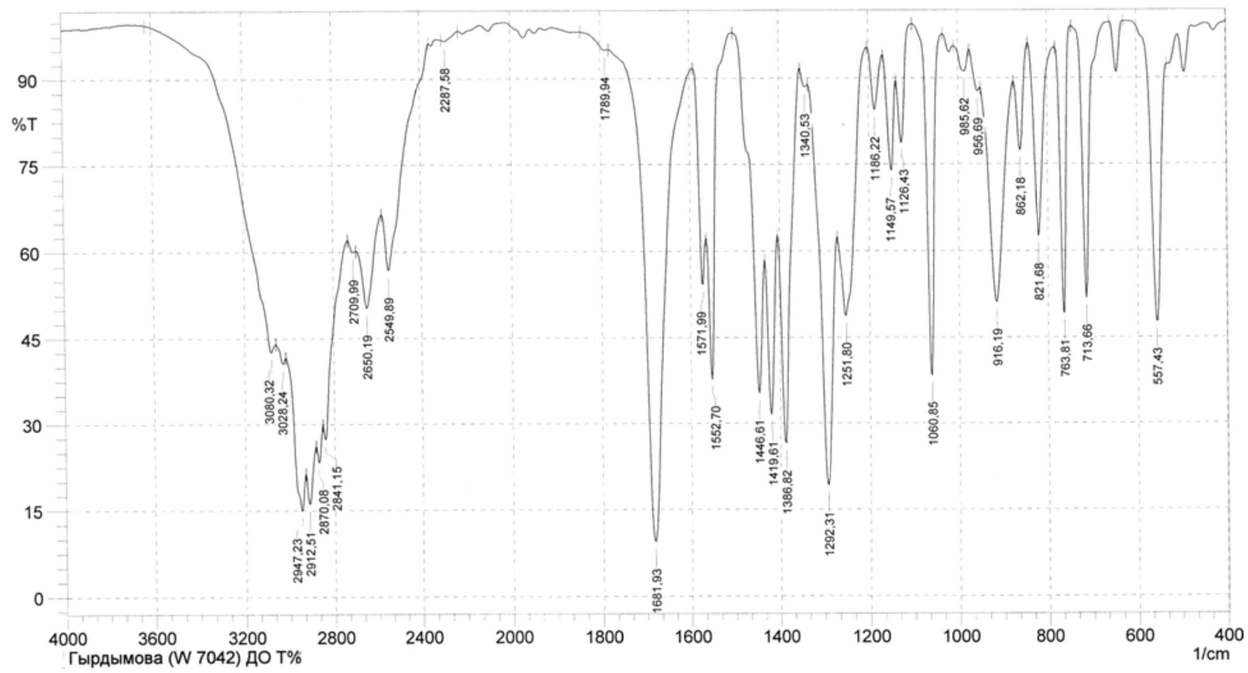
%T
3080,32 3028,24 2947,23 2912,51 2870,08 2841,15 2709,99 2650,19 2549,89 2287,58 1789,94 1681,93 1571,99 1552,70 1446,61 1419,61 1386,82 1340,53 1292,31 1251,80 1186,22 1149,57 1126,43 1060,85 985,62 956,69 916,19 862,18 821,68 763,81 713,66 557,43
1/cm
Гырдымова (W 7042) ДО T%

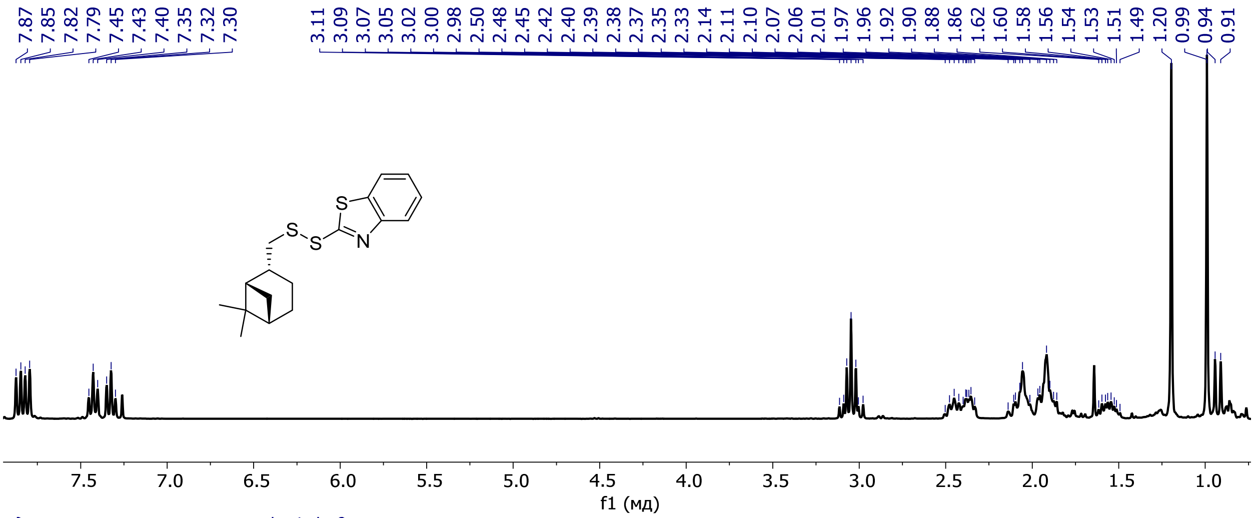
7.87 7.85 7.82 7.79 7.45 7.43 7.40 7.35 7.32 7.30
3.11 3.09 3.07 3.05 3.02 3.00 2.98 2.50 2.48 2.45 2.42 2.40 2.39 2.38 2.37 2.35 2.33 2.14 2.11 2.10 2.07 2.06 2.01 1.97 1.96 1.92 1.90 1.88 1.86 1.62 1.60 1.58 1.56 1.54 1.53 1.51 1.49 1.20 0.99 0.94 0.91
S
S
S
N
f1 (мд)

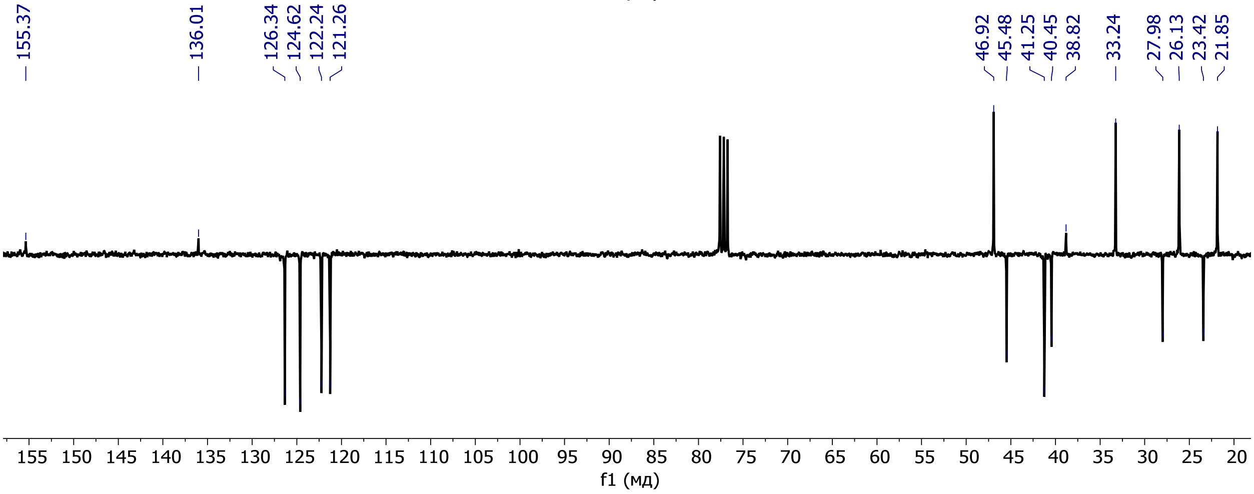
155.37 136.01 126.34 124.62 122.24 121.26 46.92 45.48 41.25 40.45 38.82 33.24 27.98 26.13 23.42 21.85
f1 (мд)

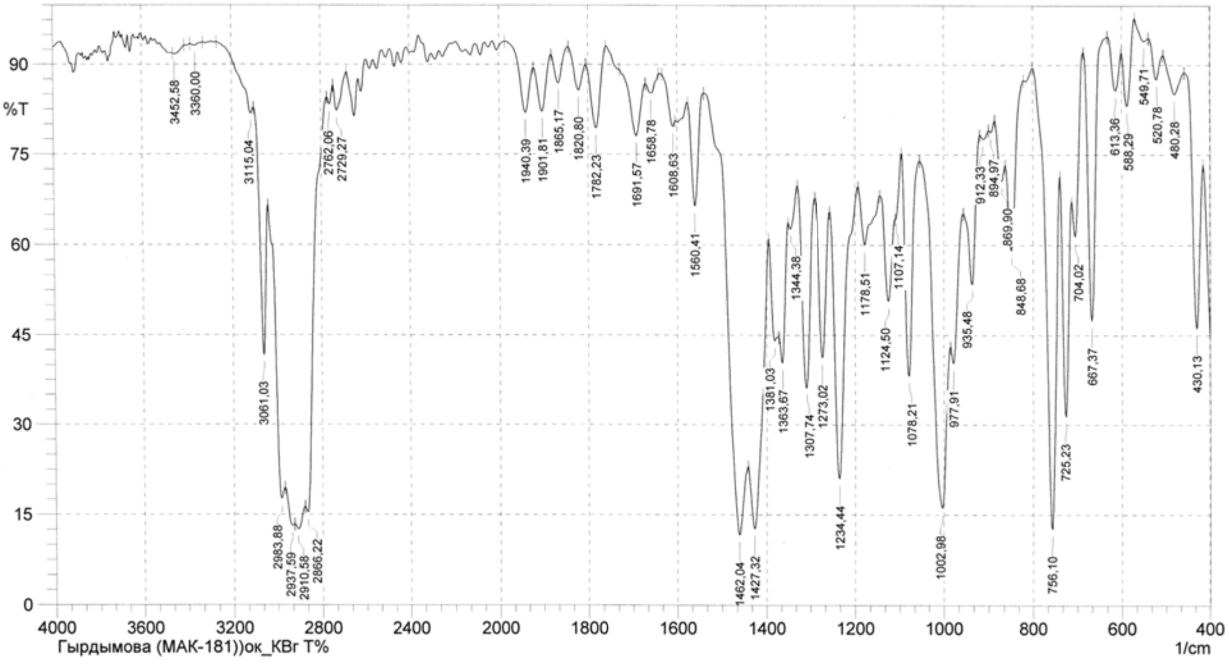
%T
3452,58 3360,00 3115,04 3061,03 2983,88 2937,59 2910,58 2866,22 2762,06 2729,27 1940,39 1901,81 1865,17 1820,80 1782,23 1691,57 1658,78 1608,63 1560,41 1462,04 1427,32 1381,03 1363,67 1344,38 1307,74 1273,02 1234,44 1178,51 1124,50 1107,14 1078,21 1002,98 977,91 935,48 912,33 894,97 869,90 848,68 756,10 725,23 704,02 667,37 613,36 588,29 549,71 520,78 480,28 430,13
1/cm
Гырдымова (MAK-181))ок_KBr T%

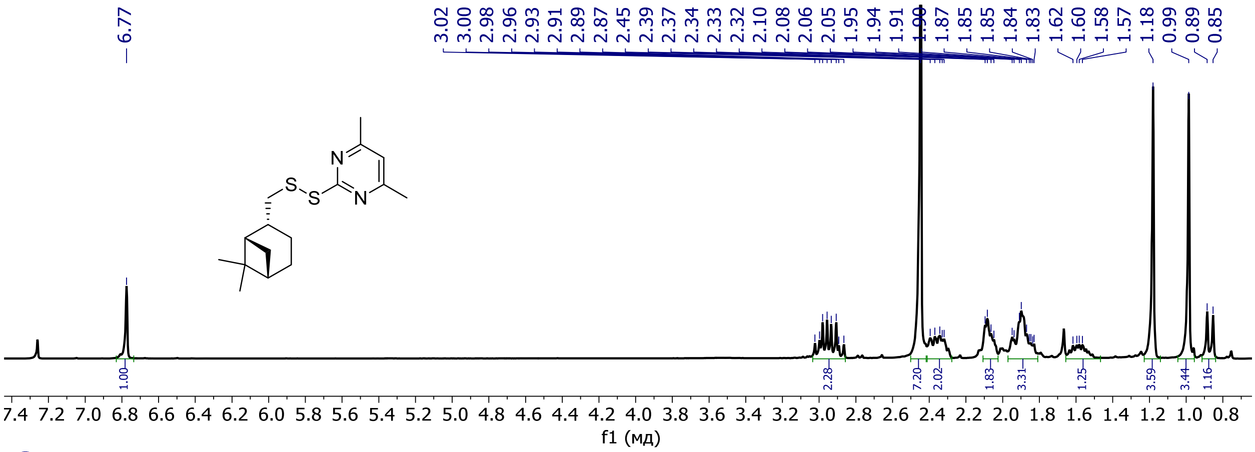
6.77
3.02 3.00 2.98 2.96 2.93 2.91 2.89 2.87 2.45 2.39 2.37 2.34 2.33 2.32 2.10 2.08 2.06 2.05 1.95 1.94 1.91 1.90 1.87 1.85 1.85 1.84 1.83 1.62 1.60 1.58 1.57 1.18 0.99 0.89 0.85
S
S
N
N
1.00
2.28
7.20
2.02
1.83
3.31
1.25
3.59
3.44
1.16
7.4 7.2 7.0 6.8 6.6 6.4 6.2 6.0 5.8 5.6 5.4 5.2 5.0 4.8 4.6 4.4 4.2 4.0 3.8 3.6 3.4 3.2 3.0 2.8 2.6 2.4 2.2 2.0 1.8 1.6 1.4 1.2 1.0 0.8
f1 (мд)

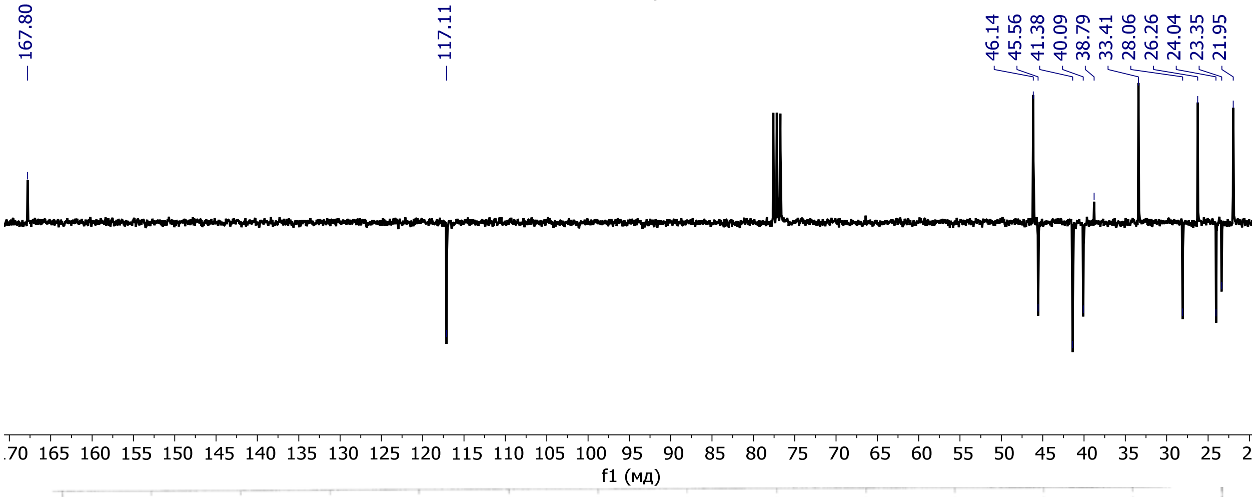
167.80
117.11
46.14 45.56 41.38 40.09 38.79 33.41 28.06 26.26 24.04 23.35 21.95
70 165 160 155 150 145 140 135 130 125 120 115 110 105 100 95 90 85 80 75 70 65 60 55 50 45 40 35 30 25
f1 (мд)

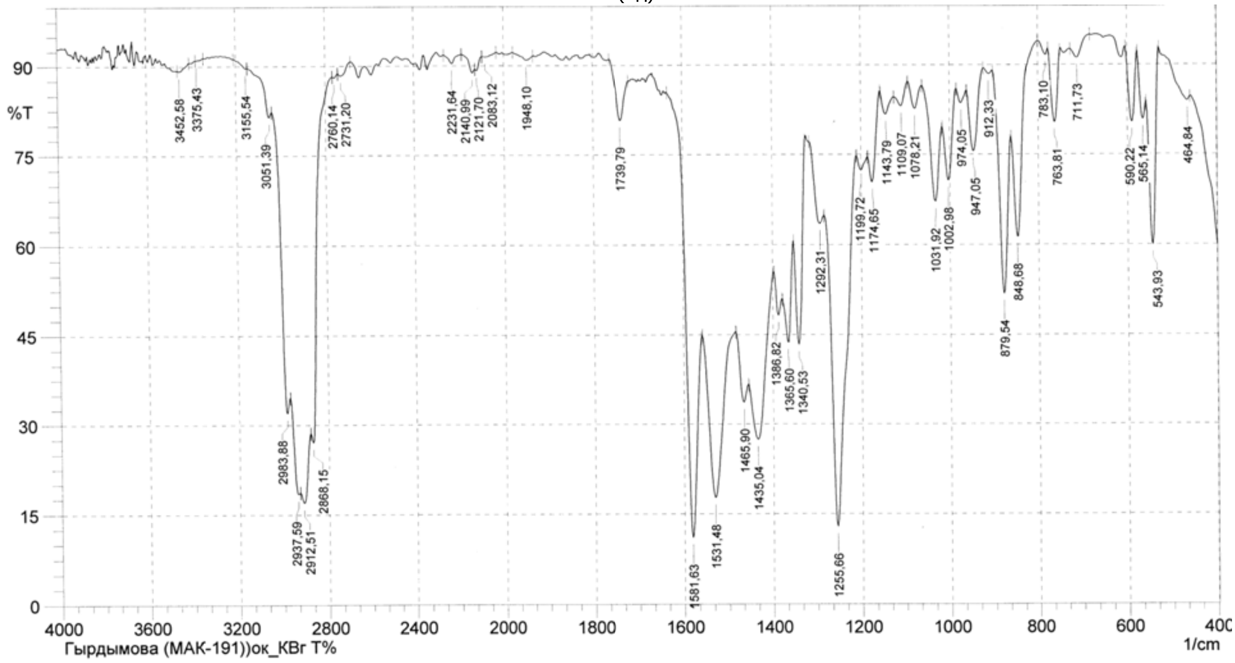
%T
90
75
60
45
30
15
0
3452,58
3375,43
3155,54
3051,39
2983,88
2937,59
2912,51
2868,15
2760,14
2731,20
2231,64
2140,99
2121,70
2083,12
1948,10
1739,79
1581,63
1531,48
1465,90
1435,04
1386,82
1365,60
1340,53
1292,31
1255,66
1199,72
1174,65
1143,79
1109,07
1078,21
1031,92
1002,98
974,05
947,05
912,33
879,54
848,68
783,10
763,81
711,73
590,22
565,14
543,93
464,84
4000 3600 3200 2800 2400 2000 1800 1600 1400 1200 1000 800 600 400
1/cm
Гырдымова (МАК-191))ок_KBr T%

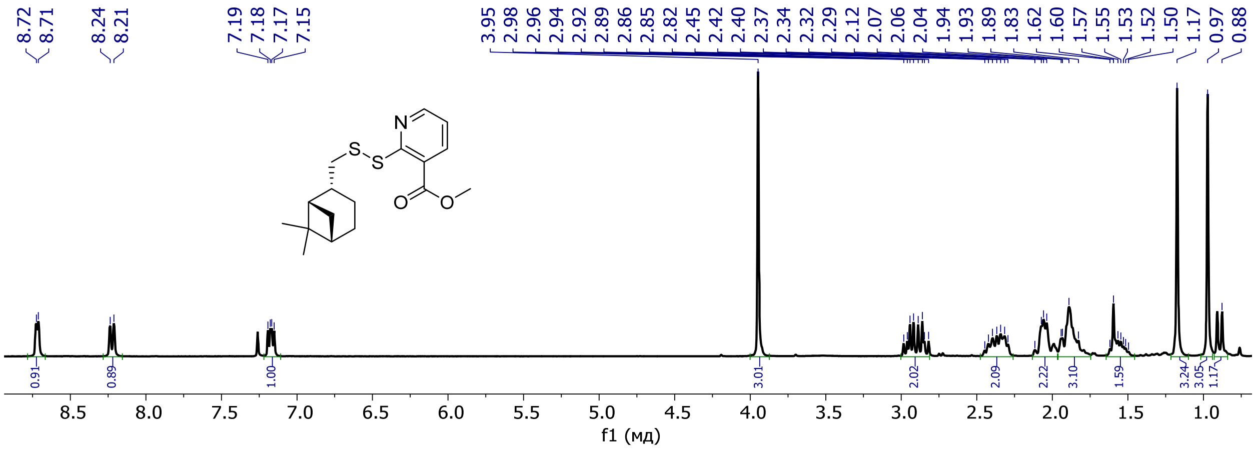
8.72
8.71
8.24
8.21
7.19
7.18
7.17
7.15
3.95
2.98
2.96
2.94
2.92
2.89
2.86
2.85
2.82
2.45
2.42
2.40
2.37
2.34
2.32
2.29
2.12
2.07
2.06
2.04
1.94
1.93
1.89
1.83
1.62
1.60
1.57
1.55
1.53
1.52
1.50
1.17
0.97
0.88
N
S
S
O
O
0.91
0.89
1.00
3.01
2.02
2.09
2.22
3.10
1.59
3.24
3.05
1.17
8.5 8.0 7.5 7.0 6.5 6.0 5.5 5.0 4.5 4.0 3.5 3.0 2.5 2.0 1.5 1.0
f1 (мд)

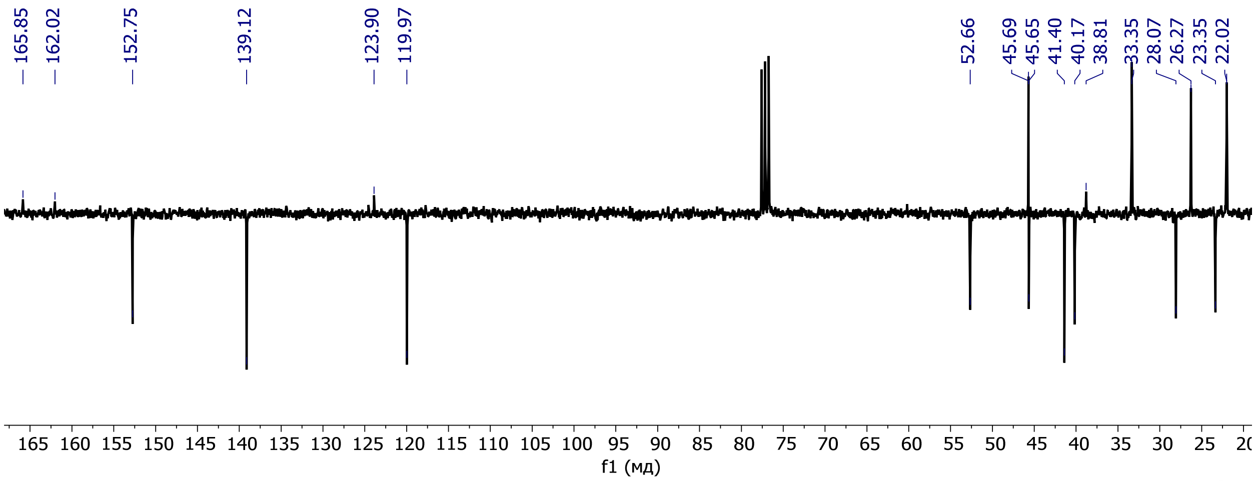
165.85
162.02
152.75
139.12
123.90
119.97
52.66
45.69
45.65
41.40
40.17
38.81
33.35
28.07
26.27
23.35
22.02
165 160 155 150 145 140 135 130 125 120 115 110 105 100 95 90 85 80 75 70 65 60 55 50 45 40 35 30 25 20
f1 (мд)

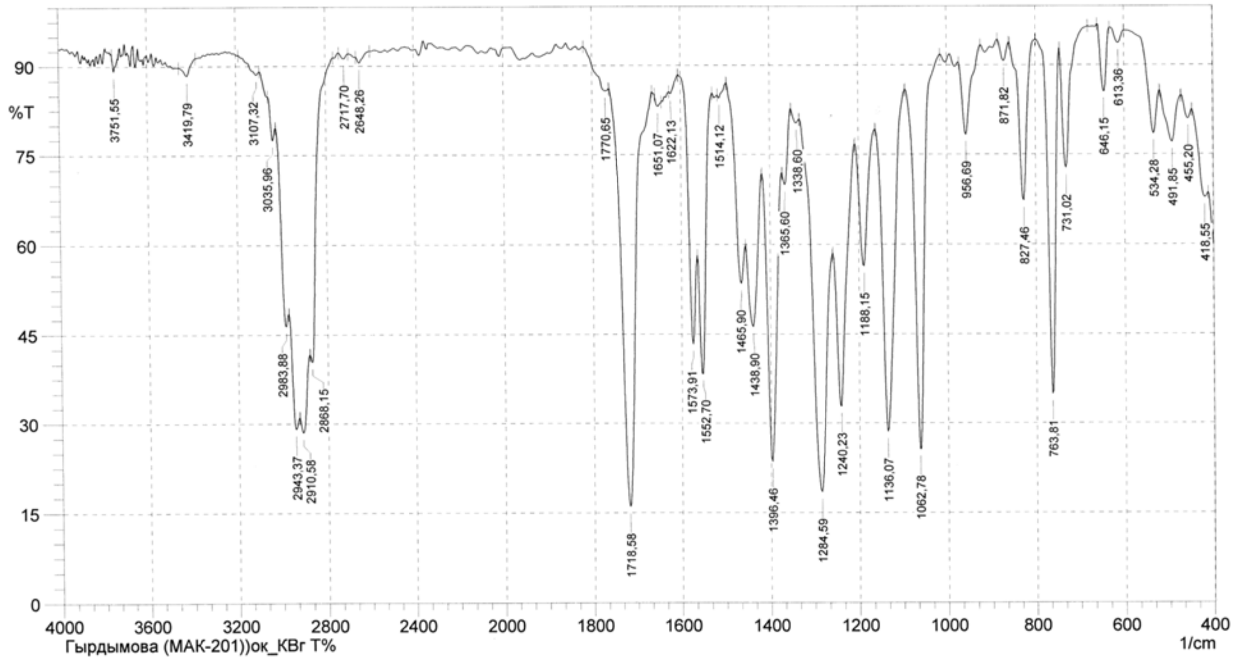
90
%T
75
60
45
30
15
0
3751,55
3419,79
3107,32
3035,96
2983,88
2943,37
2910,58
2868,15
2717,70
2648,26
1770,65
1718,58
1651,07
1622,13
1573,91
1552,70
1514,12
1465,90
1438,90
1396,46
1365,60
1338,60
1284,59
1240,23
1188,15
1136,07
1062,78
956,69
871,82
827,46
763,81
731,02
646,15
613,36
534,28
491,85
455,20
418,55
4000 3600 3200 2800 2400 2000 1800 1600 1400 1200 1000 800 600 400
1/cm
Гырдымова (МАК-201))ок_KBr T%

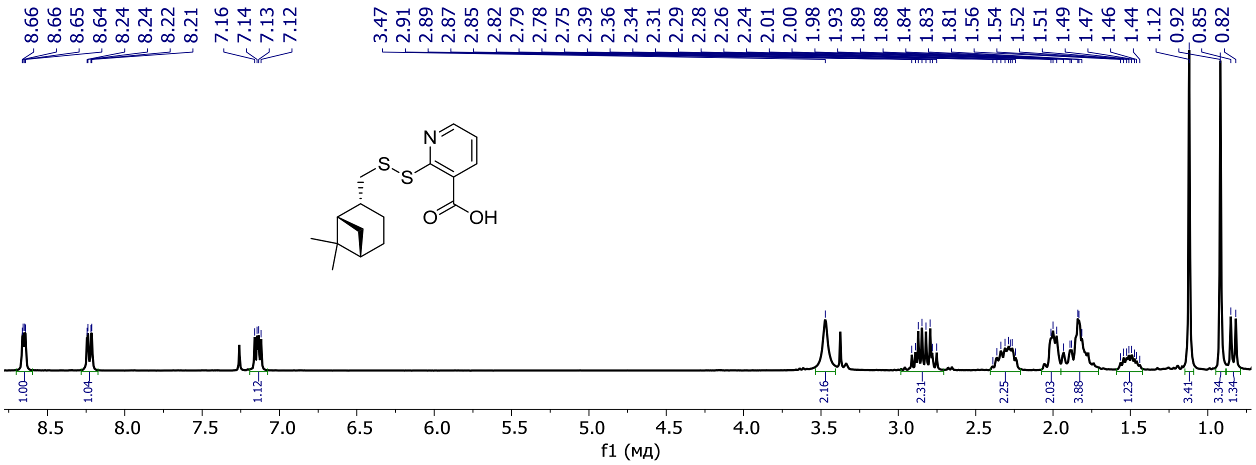
8.66 8.66 8.65 8.64 8.24 8.24 8.22 8.21 7.16 7.14 7.13 7.12
3.47 2.91 2.89 2.87 2.85 2.82 2.79 2.78 2.75 2.39 2.36 2.34 2.31 2.29 2.28 2.26 2.24 2.01 2.00 1.98 1.93 1.89 1.88 1.84 1.83 1.81 1.56 1.54 1.52 1.51 1.49 1.47 1.46 1.44 1.12 0.92 0.85 0.82
N
S
S
O
OH
1.00 1.04 1.12 2.16 2.31 2.25 2.03 3.88 1.23 3.41 3.34 1.34
8.5 8.0 7.5 7.0 6.5 6.0 5.5 5.0 4.5 4.0 3.5 3.0 2.5 2.0 1.5 1.0
f1 (мд)

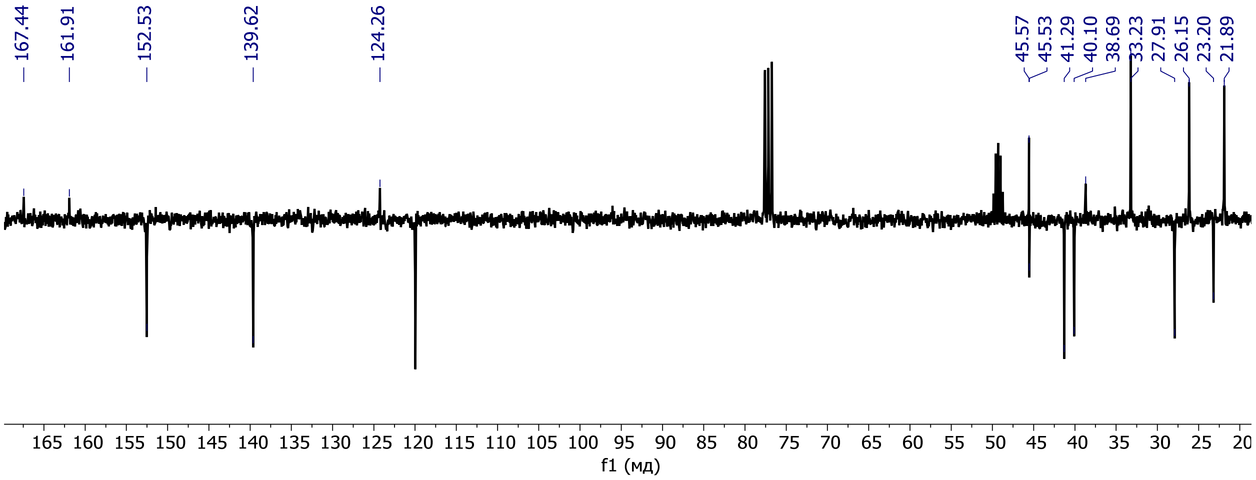
167.44 161.91 152.53 139.62 124.26
45.57 45.53 41.29 40.10 38.69 33.23 27.91 26.15 23.20 21.89
165 160 155 150 145 140 135 130 125 120 115 110 105 100 95 90 85 80 75 70 65 60 55 50 45 40 35 30 25 20
f1 (мд)

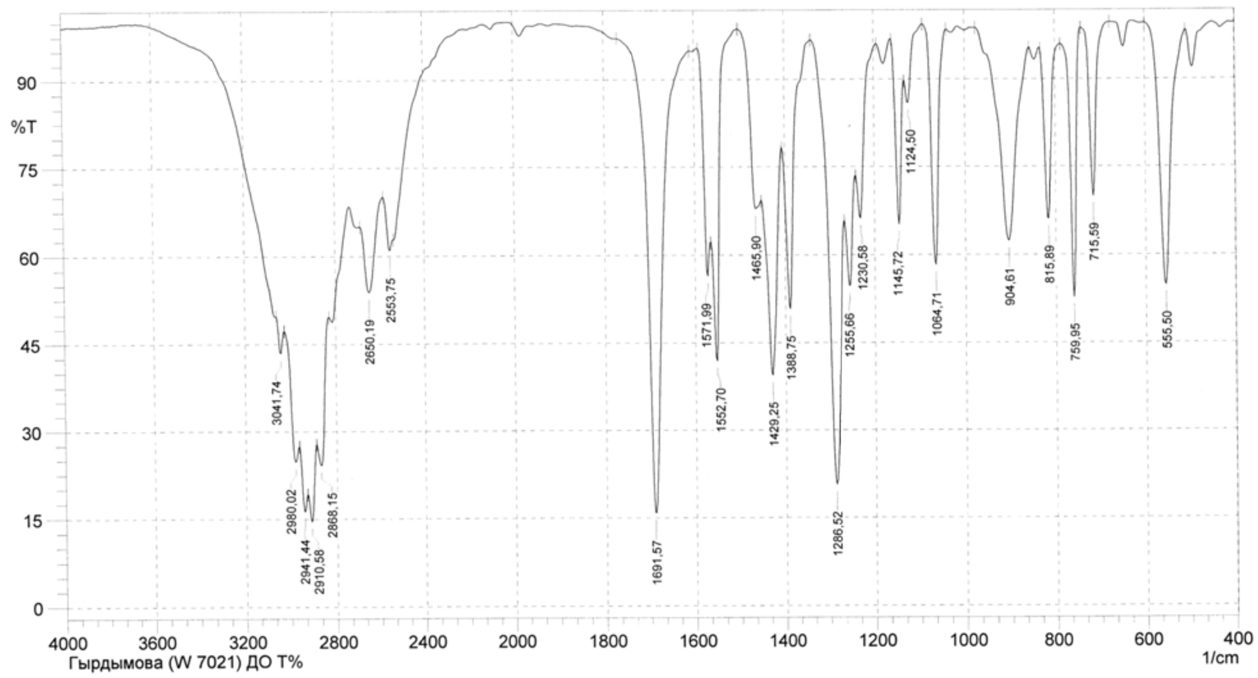
90
%T
75
60
45
30
15
0
3041,74
2980,02
2941,44
2910,58
2868,15
2650,19
2553,75
1691,57
1571,99
1552,70
1465,90
1429,25
1388,75
1286,52
1255,66
1230,58
1145,72
1124,50
1064,71
904,61
815,89
759,95
715,59
555,50
4000 3600 3200 2800 2400 2000 1800 1600 1400 1200 1000 800 600 400
1/cm
Гырдымова (W 7021) ДО Т%

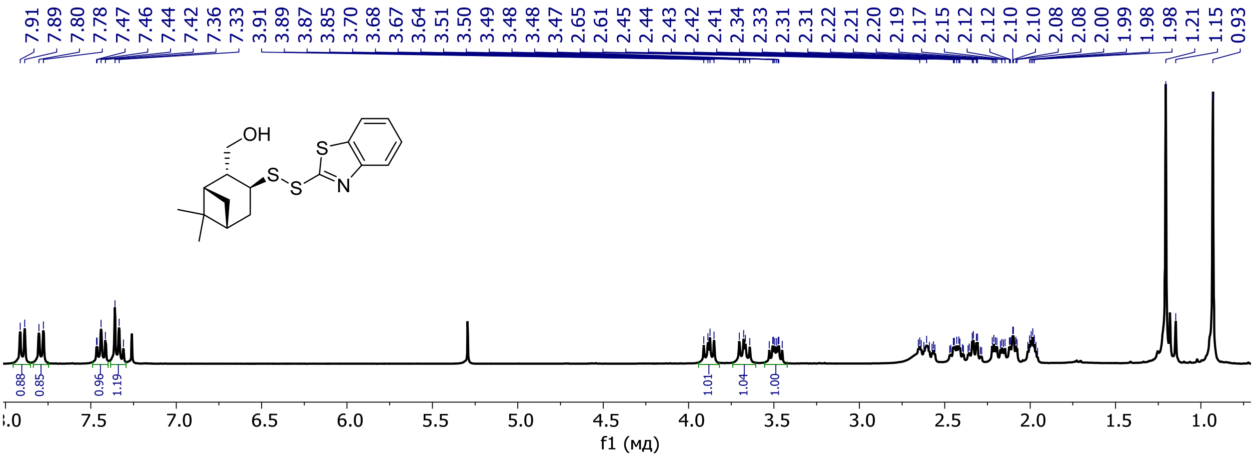
7.91 7.89 7.80 7.78 7.47 7.46 7.44 7.42 7.36 7.33 3.91 3.89 3.87 3.85 3.70 3.68 3.67 3.64 3.51 3.50 3.49 3.48 3.48 3.47 2.65 2.61 2.45 2.44 2.43 2.42 2.41 2.34 2.33 2.31 2.31 2.22 2.21 2.20 2.19 2.17 2.15 2.12 2.12 2.10 2.10 2.08 2.08 2.00 1.99 1.98 1.98 1.21 1.15 0.93
OH
S
S
S
N
0.88 0.85 0.96 1.19 1.01 1.04 1.00
f1 (мд)

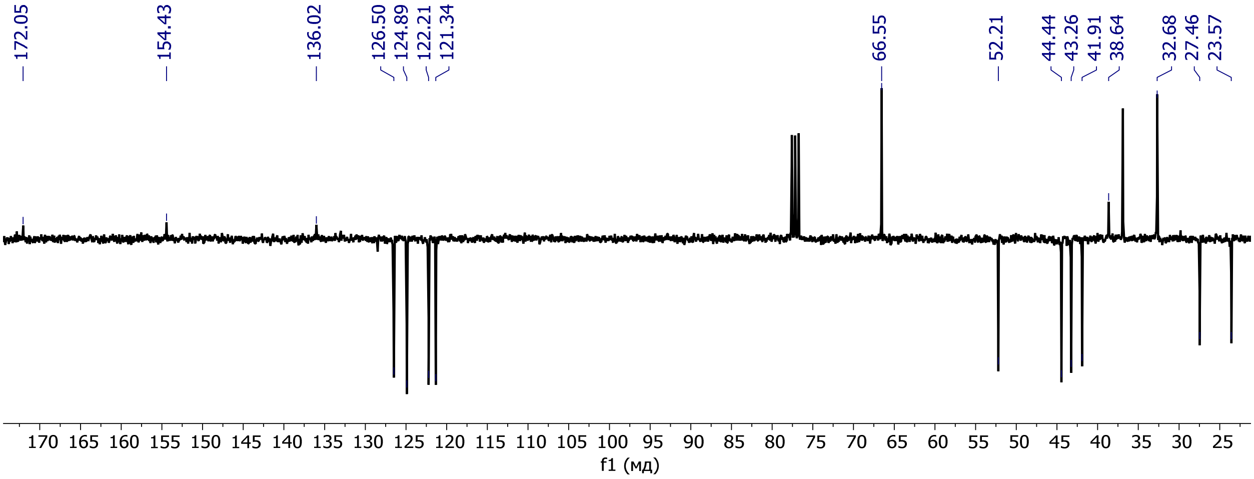
172.05 154.43 136.02 126.50 124.89 122.21 121.34 66.55 52.21 44.44 43.26 41.91 38.64 32.68 27.46 23.57
f1 (мд)

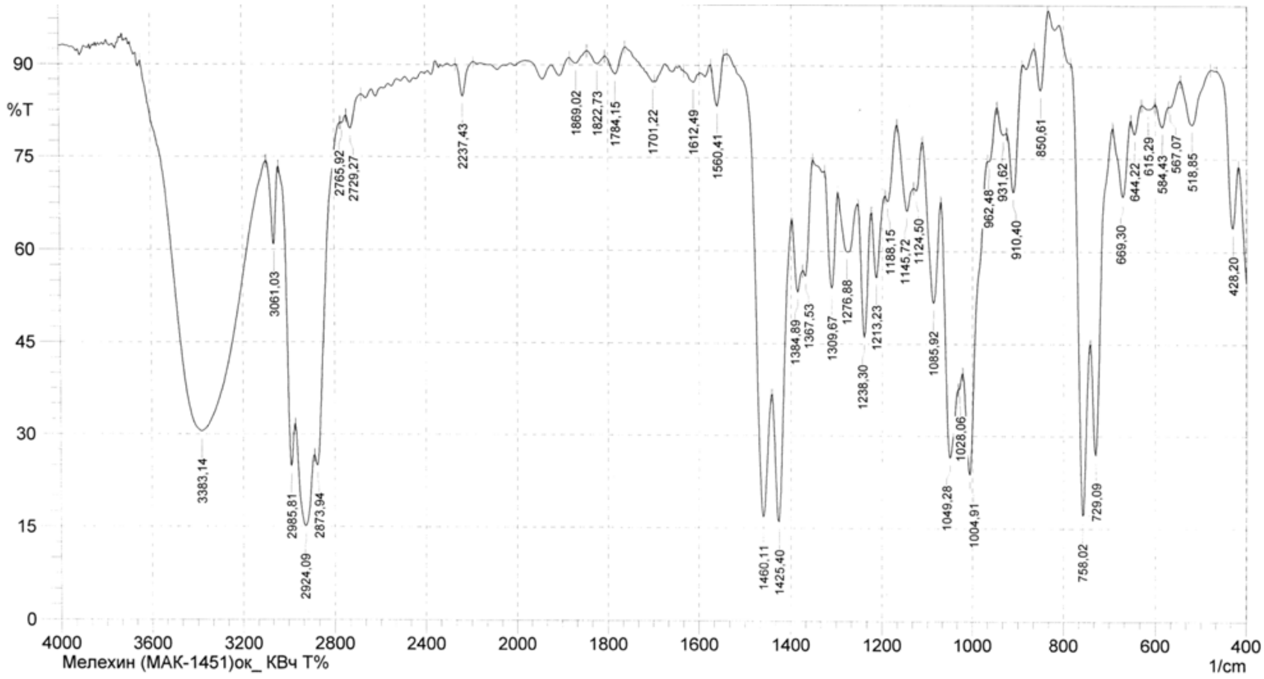
%T
3383,14 3061,03 2985,81 2924,09 2873,94 2765,92 2729,27 2237,43 1869,02 1822,73 1784,15 1701,22 1612,49 1560,41 1460,11 1425,40 1384,89 1367,53 1309,67 1276,88 1238,30 1213,23 1188,15 1145,72 1124,50 1085,92 1049,28 1028,06 1004,91 962,48 931,62 910,40 850,61 758,02 729,09 669,30 644,22 615,29 584,43 567,07 518,85 428,20
Мелехин (МАК-1451)ок_ КВч T%
1/cm

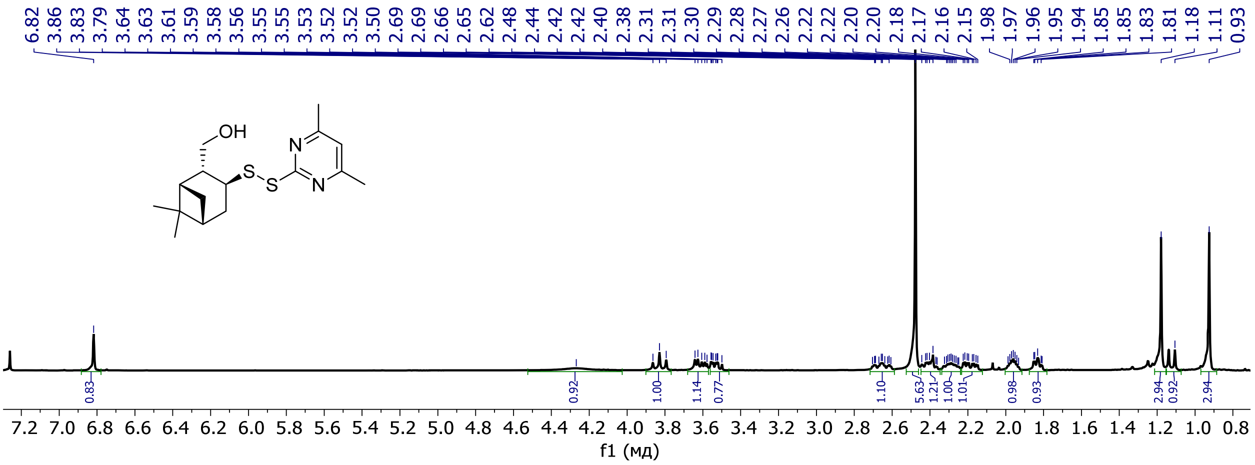
6.82
3.86
3.83
3.79
3.64
3.63
3.61
3.59
3.58
3.56
3.55
3.55
3.53
3.52
3.52
3.50
2.69
2.69
2.66
2.65
2.62
2.48
2.44
2.42
2.42
2.40
2.38
2.31
2.31
2.30
2.29
2.28
2.27
2.26
2.22
2.22
2.20
2.20
2.18
2.17
2.16
2.15
1.98
1.97
1.96
1.95
1.94
1.85
1.85
1.83
1.81
1.18
1.11
0.93
OH
S
S
N
N
0.83
0.92
1.00
1.14
0.77
1.10
5.63
1.21
1.00
1.01
0.98
0.93
2.94
0.92
2.94
7.2 7.0 6.8 6.6 6.4 6.2 6.0 5.8 5.6 5.4 5.2 5.0 4.8 4.6 4.4 4.2 4.0 3.8 3.6 3.4 3.2 3.0 2.8 2.6 2.4 2.2 2.0 1.8 1.6 1.4 1.2 1.0 0.8
f1 (мд)

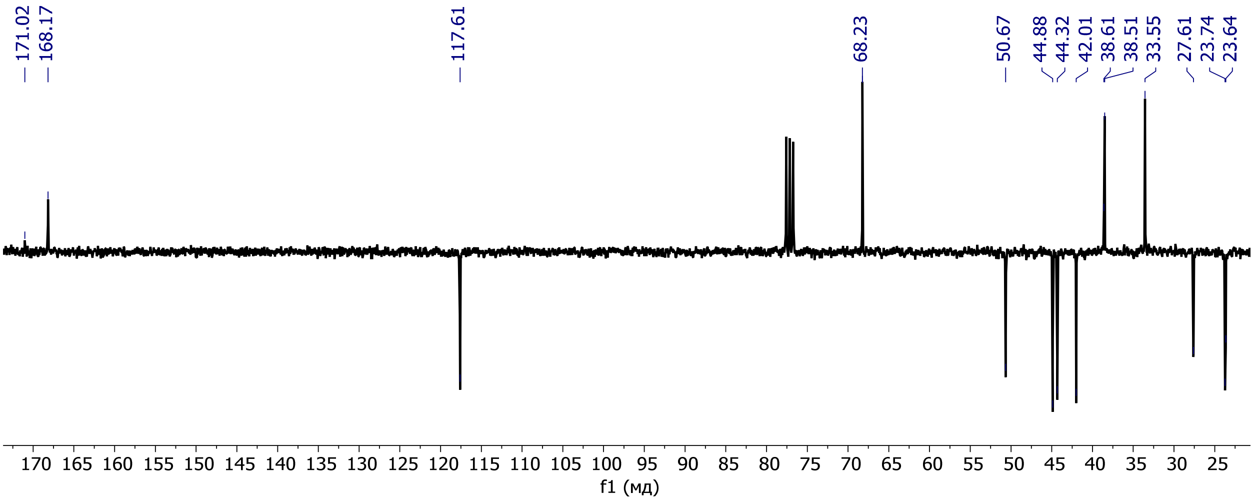
171.02
168.17
117.61
68.23
50.67
44.88
44.32
42.01
38.61
38.51
33.55
27.61
23.74
23.64
170 165 160 155 150 145 140 135 130 125 120 115 110 105 100 95 90 85 80 75 70 65 60 55 50 45 40 35 30 25
f1 (мд)

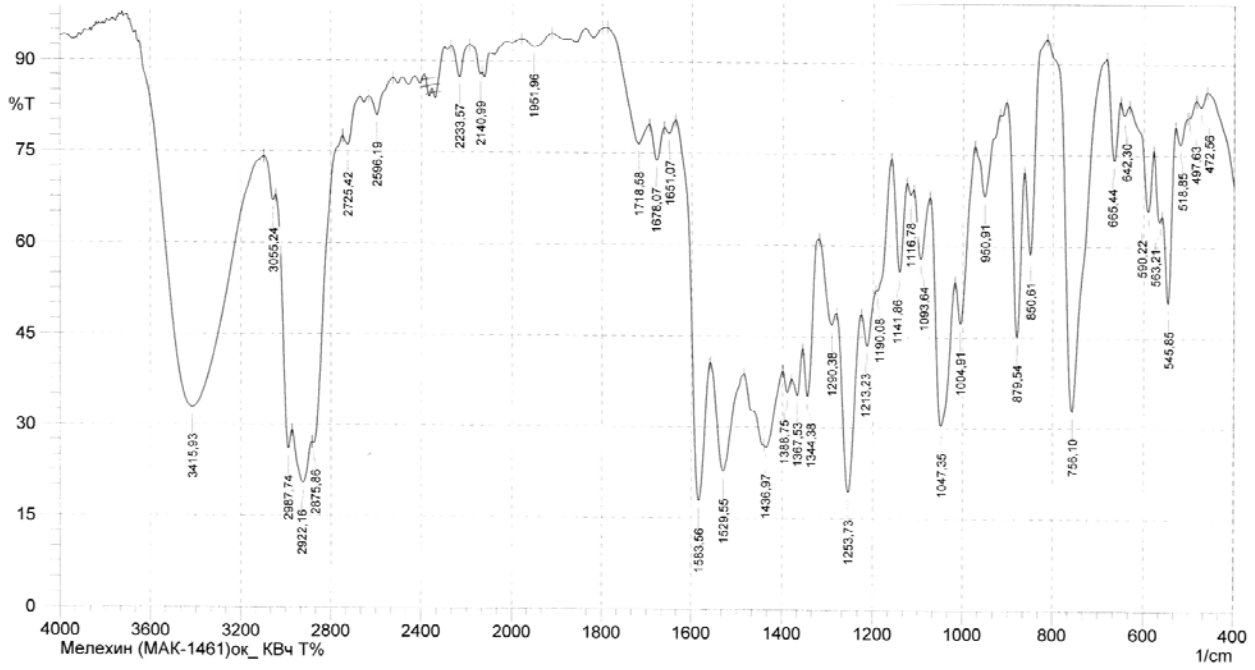
%T
90
75
60
45
30
15
0
3415,93
3055,24
2987,74
2922,16
2875,86
2725,42
2596,19
2233,57
2140,99
1951,96
1718,58
1676,07
1651,07
1583,56
1529,55
1436,97
1388,75
1367,53
1344,38
1290,38
1253,73
1213,23
1190,08
1141,86
1116,78
1093,64
1047,35
1004,91
950,91
879,54
850,61
756,10
665,44
642,30
590,22
563,21
545,85
518,85
497,63
472,56
4000 3600 3200 2800 2400 2000 1800 1600 1400 1200 1000 800 600 400
1/cm
Мелехин (МАК-1461)ок_ КВч T%

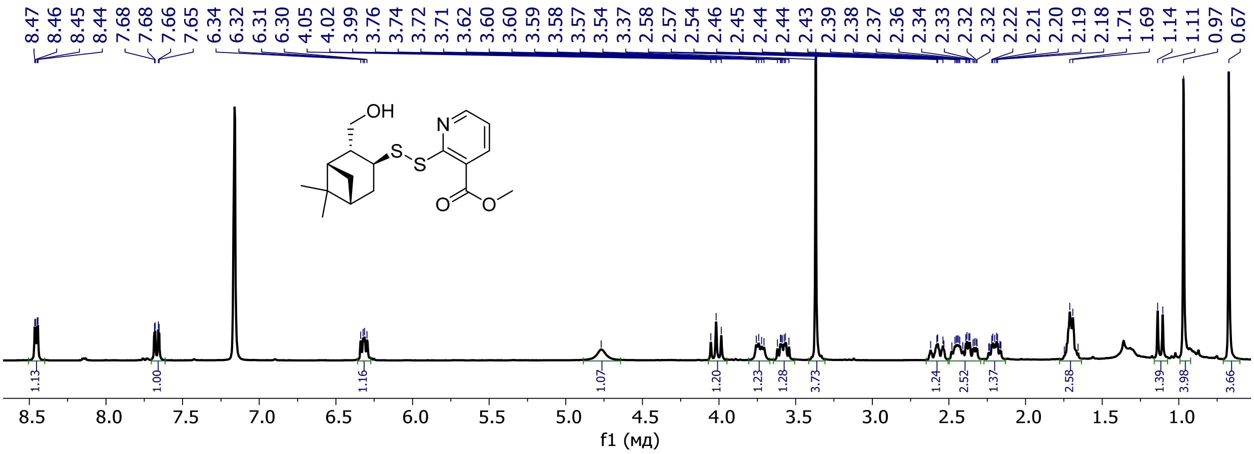
8.47 8.46 8.45 8.44 7.68 7.66 7.65 6.34 6.32 6.31 6.30 4.05 4.02 3.99 3.76 3.74 3.72 3.71 3.62 3.60 3.60 3.59 3.58 3.57 3.54 3.37 2.58 2.57 2.54 2.46 2.45 2.44 2.44 2.43 2.39 2.38 2.37 2.36 2.34 2.33 2.32 2.32 2.22 2.21 2.20 2.19 2.18 1.71 1.69 1.14 1.11 0.97 0.67
OH
N
S
S
O
O
1.13 1.00 1.16 1.07 1.20 1.23 1.28 3.73 1.24 2.52 1.37 2.58 1.39 3.98 3.66
8.5 8.0 7.5 7.0 6.5 6.0 5.5 5.0 4.5 4.0 3.5 3.0 2.5 2.0 1.5 1.0
f1 (мд)

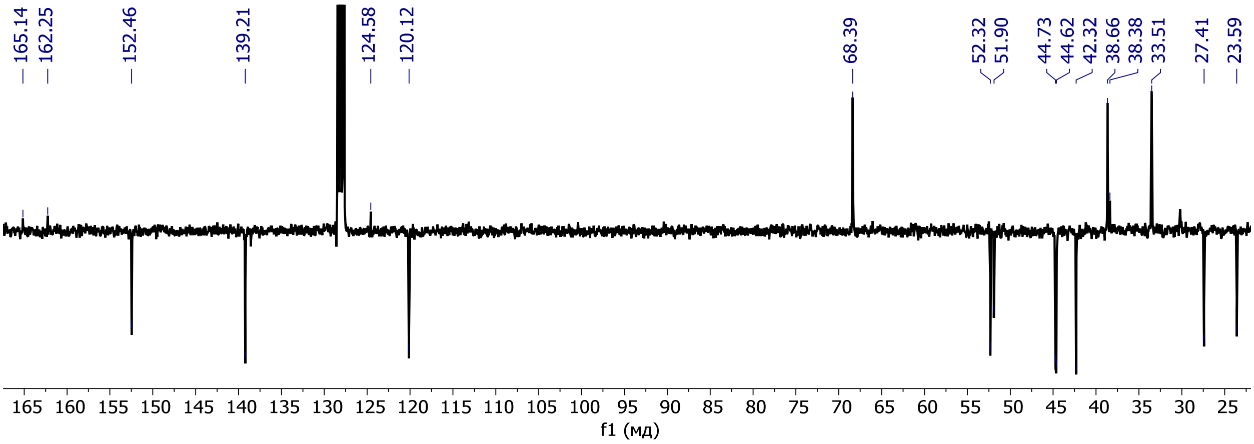
165.14 162.25 152.46 139.21 124.58 120.12 68.39 52.32 51.90 44.73 44.62 42.32 38.66 38.38 33.51 27.41 23.59
165 160 155 150 145 140 135 130 125 120 115 110 105 100 95 90 85 80 75 70 65 60 55 50 45 40 35 30 25
f1 (мд)

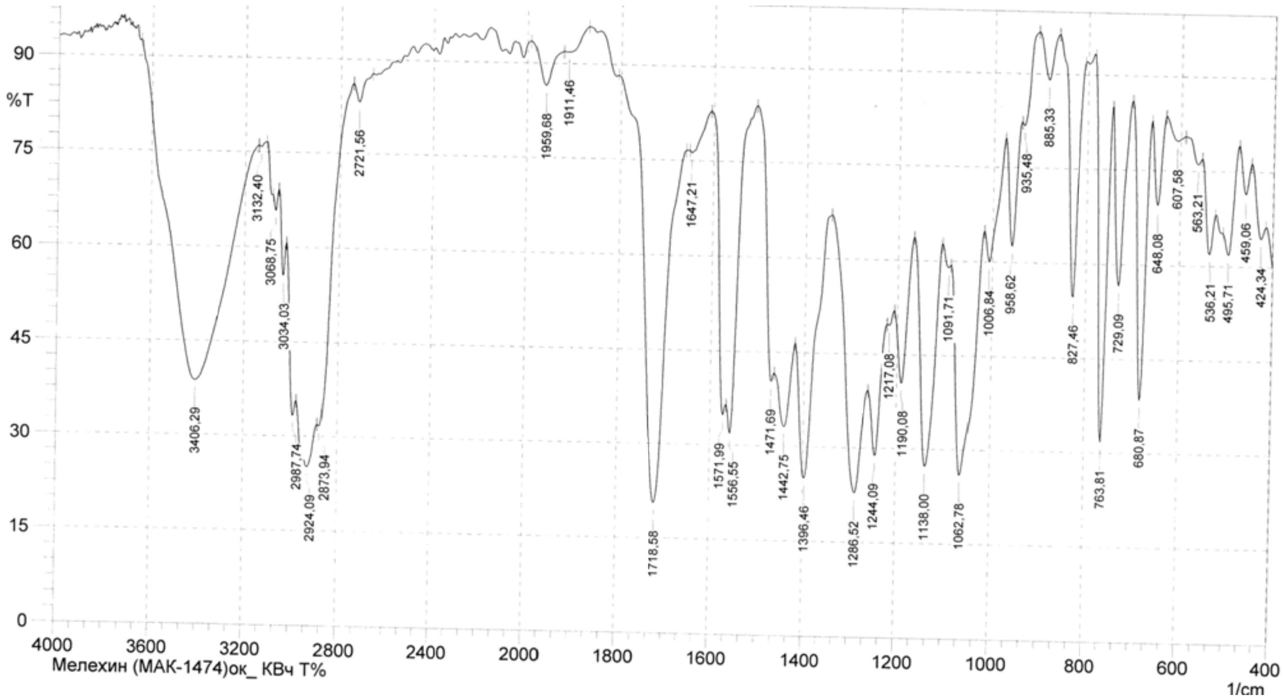
%T
90
75
60
45
30
15
0
3406,29 3132,40 3068,75 3034,03 2987,74 2924,09 2873,94 2721,56 1959,68 1911,46 1718,58 1647,21 1571,99 1556,55 1471,69 1442,75 1396,46 1286,52 1244,09 1217,08 1190,08 1138,00 1091,71 1062,78 1006,84 958,62 935,48 885,33 827,46 763,81 729,09 680,87 648,08 607,58 563,21 536,21 495,71 459,06 424,34
4000 3600 3200 2800 2400 2000 1800 1600 1400 1200 1000 800 600 400
1/cm
Мелехин (МАК-1474)ок_ КВч T%

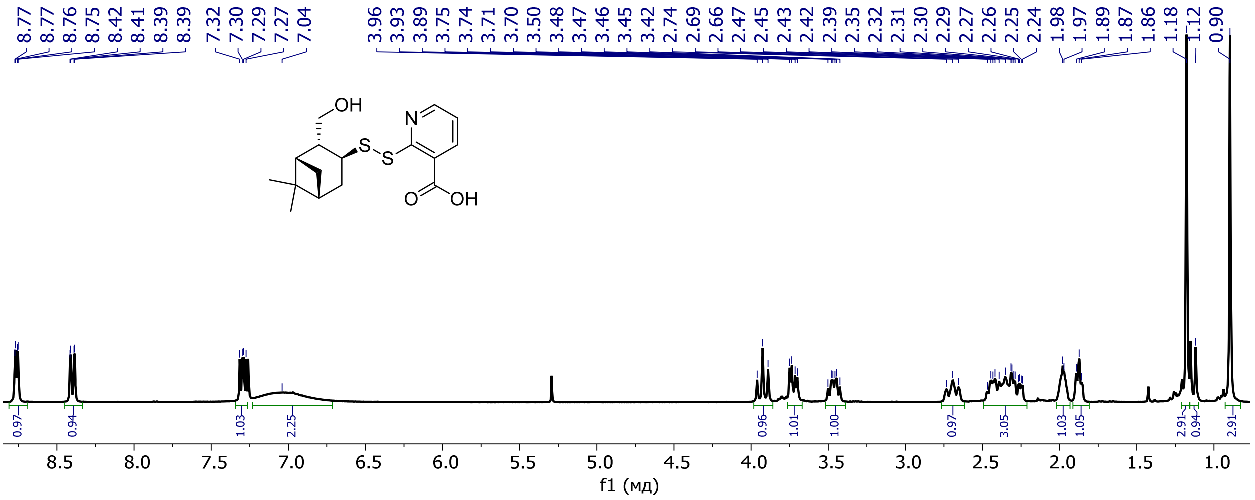
8.77 8.77 8.76 8.75 8.42 8.41 8.39 8.39 7.32 7.30 7.29 7.27 7.04
3.96 3.93 3.89 3.75 3.74 3.71 3.70 3.50 3.48 3.47 3.46 3.45 3.42 2.74 2.69 2.66 2.47 2.45 2.43 2.42 2.39 2.35 2.32 2.31 2.30 2.29 2.27 2.26 2.25 2.24 1.98 1.97 1.89 1.87 1.86 1.18 1.12 0.90
OH
N
S
S
O
OH
0.97 0.94 1.03 2.25 0.96 1.01 1.00 0.97 3.05 1.03 1.05 2.91 0.94 2.91
8.5 8.0 7.5 7.0 6.5 6.0 5.5 5.0 4.5 4.0 3.5 3.0 2.5 2.0 1.5 1.0
f1 (мд)

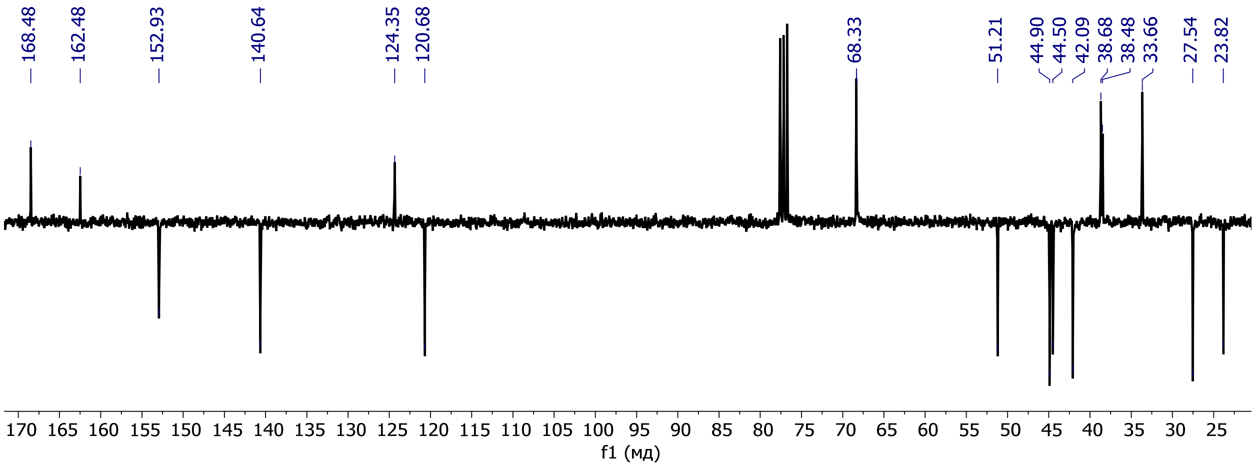
168.48 162.48 152.93 140.64 124.35 120.68 68.33 51.21 44.90 44.50 42.09 38.68 38.48 33.66 27.54 23.82
170 165 160 155 150 145 140 135 130 125 120 115 110 105 100 95 90 85 80 75 70 65 60 55 50 45 40 35 30 25
f1 (мд)

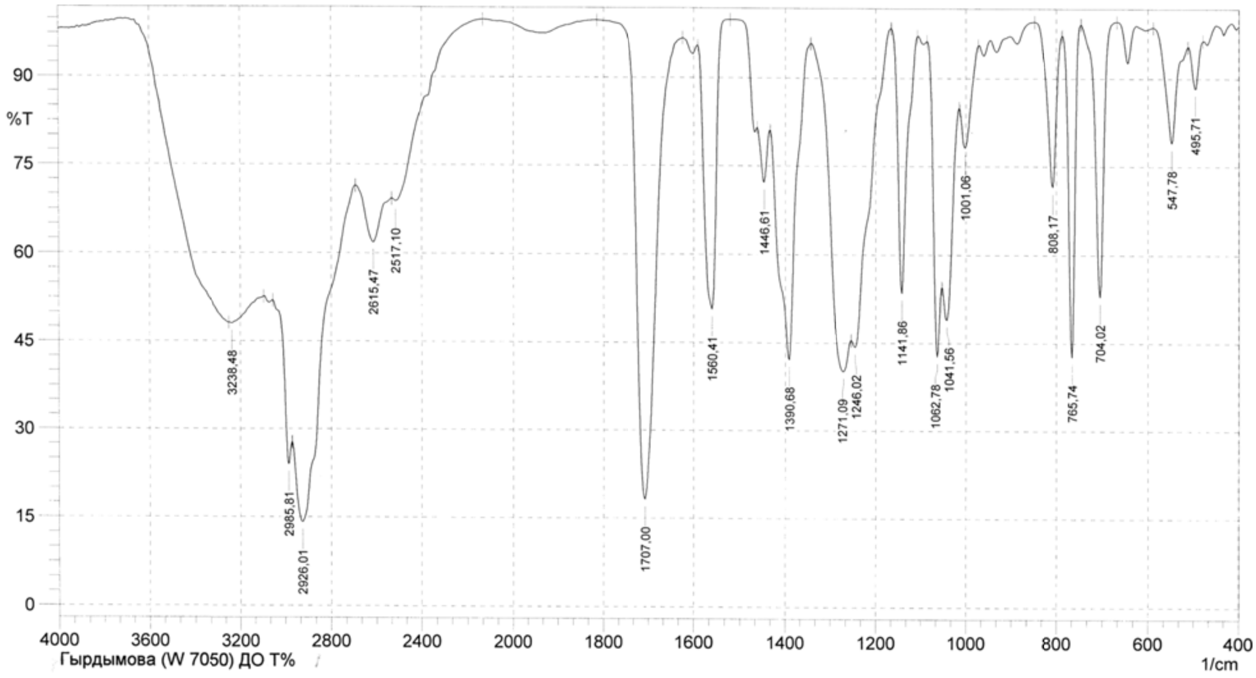
%T
90 75 60 45 30 15 0
3238,48 2985,81 2926,01 2615,47 2517,10 1707,00 1560,41 1446,61 1390,68 1271,09 1246,02 1141,86 1062,78 1041,56 1001,06 808,17 765,74 704,02 547,78 495,71
4000 3600 3200 2800 2400 2000 1800 1600 1400 1200 1000 800 600 400
1/cm
Гырдымова (W 7050) ДО T%

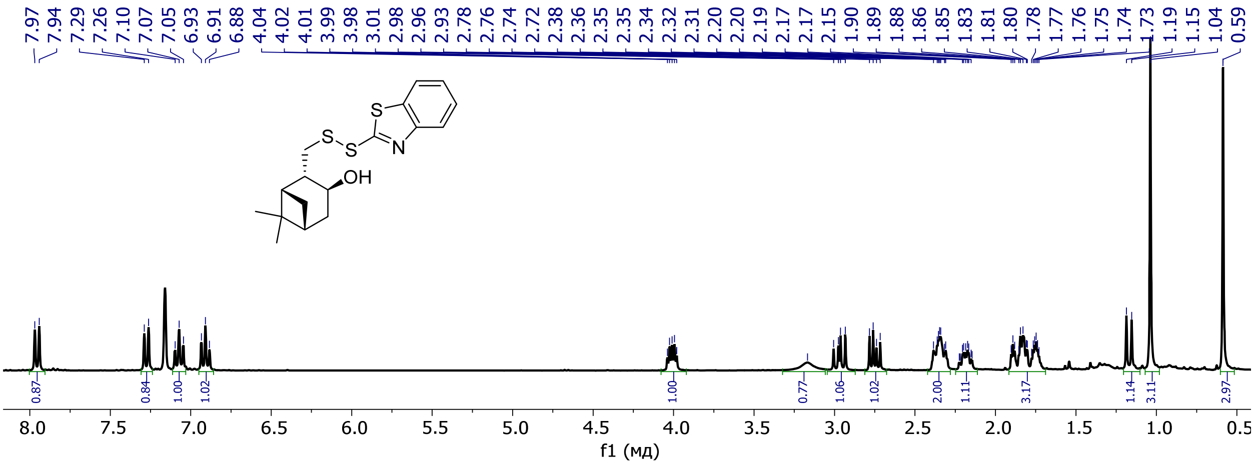
7.97 7.94 7.29 7.26 7.10 7.07 7.05 6.93 6.91 6.88 4.04 4.02 4.01 3.99 3.98 3.01 2.98 2.96 2.93 2.78 2.76 2.74 2.72 2.38 2.36 2.35 2.35 2.34 2.32 2.31 2.20 2.20 2.19 2.17 2.17 2.15 1.90 1.89 1.88 1.86 1.85 1.83 1.81 1.80 1.78 1.77 1.76 1.75 1.74 1.73 1.19 1.15 1.04 0.59
S
S
S
N
OH
0.87 0.84 1.00 1.02 1.00 0.77 1.06 1.02 2.00 1.11 3.17 1.14 3.11 2.97
8.0 7.5 7.0 6.5 6.0 5.5 5.0 4.5 4.0 3.5 3.0 2.5 2.0 1.5 1.0 0.5
f1 (мд)

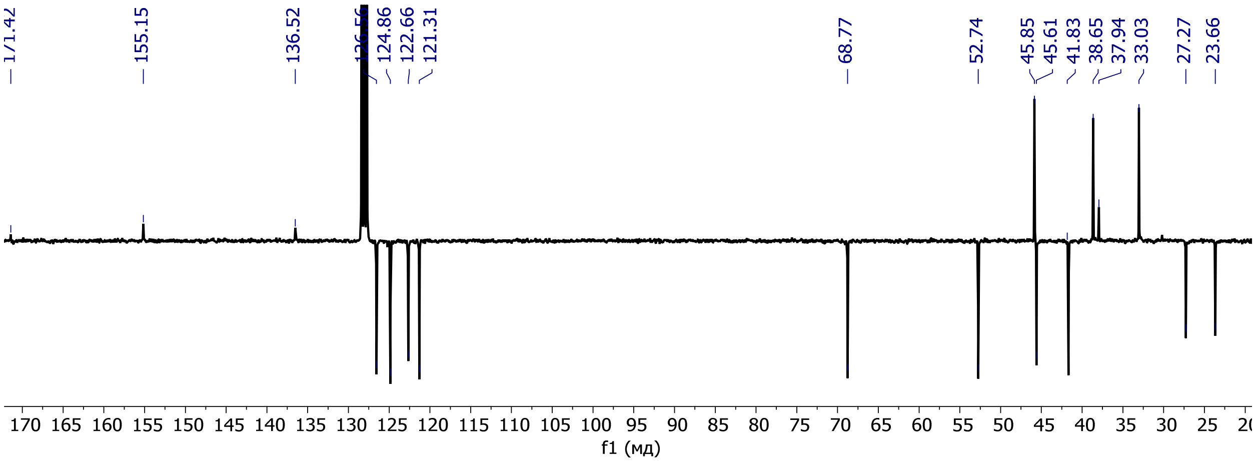
155.15
136.52
124.86
122.66
121.31
68.77
52.74
45.85
45.61
41.83
38.65
37.94
33.03
27.27
23.66
170 165 160 155 150 145 140 135 130 125 120 115 110 105 100 95 90 85 80 75 70 65 60 55 50 45 40 35 30 25
f1 (мд)

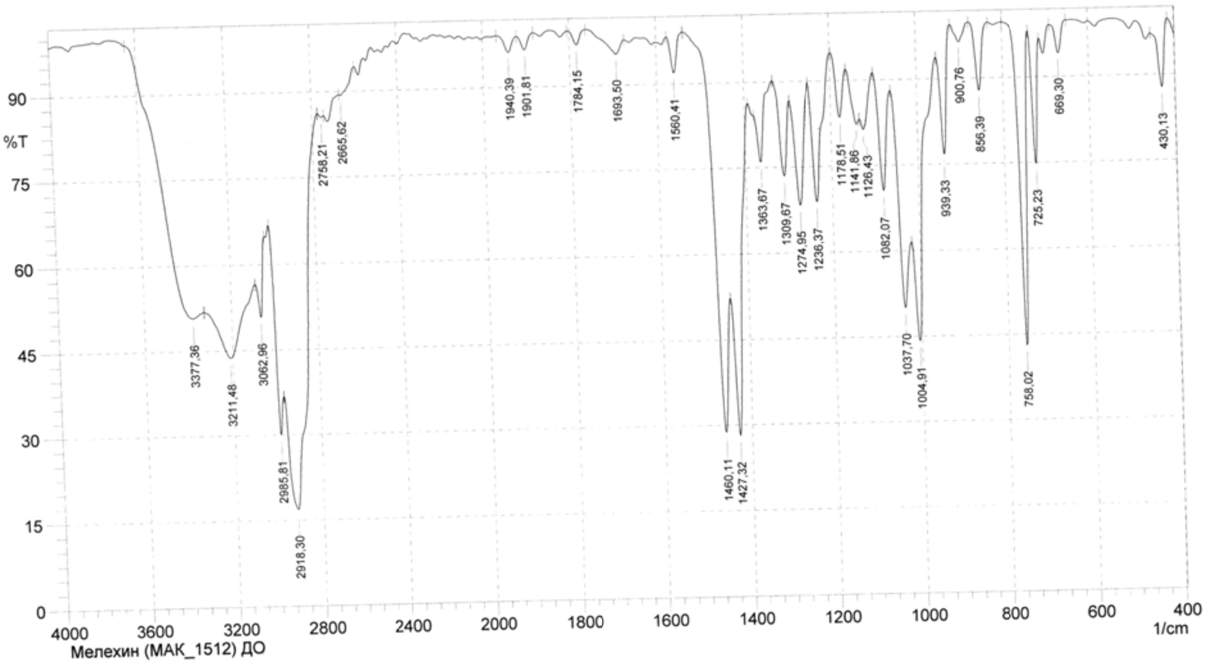
%T
90 75 60 45 30 15 0
3377,36 3211,48 3062,96 2985,81 2918,30 2758,21 2665,62 1940,39 1901,81 1784,15 1693,50 1560,41 1460,11 1427,32 1363,67 1309,67 1274,95 1236,37 1178,51 1141,86 1126,43 1082,07 1037,70 1004,91 939,33 900,76 856,39 758,02 725,23 669,30 430,13
4000 3600 3200 2800 2400 2000 1800 1600 1400 1200 1000 800 600 400
1/cm
Мелехин (МАК_1512) ДО

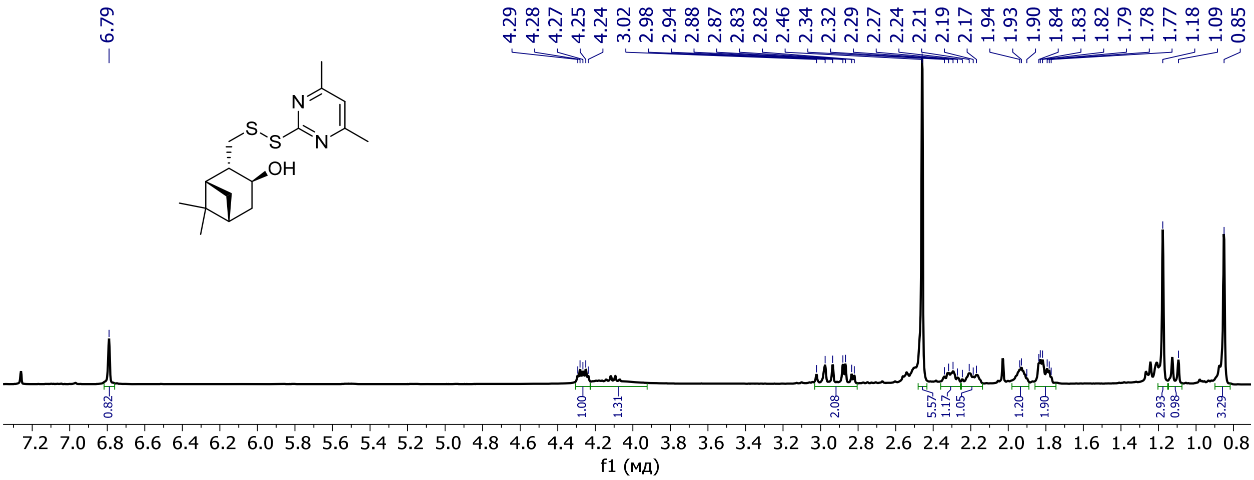
6.79
4.29 4.28 4.27 4.25 4.24 3.02 2.98 2.94 2.88 2.87 2.83 2.82 2.46 2.34 2.32 2.29 2.27 2.24 2.21 2.19 2.17 1.94 1.93 1.90 1.84 1.83 1.82 1.79 1.78 1.77 1.18 1.09 0.85
N
N
S
S
OH
0.82
1.00
1.31
2.08
5.57
1.17
1.05
1.20
1.90
2.93
0.98
3.29
f1 (мд)

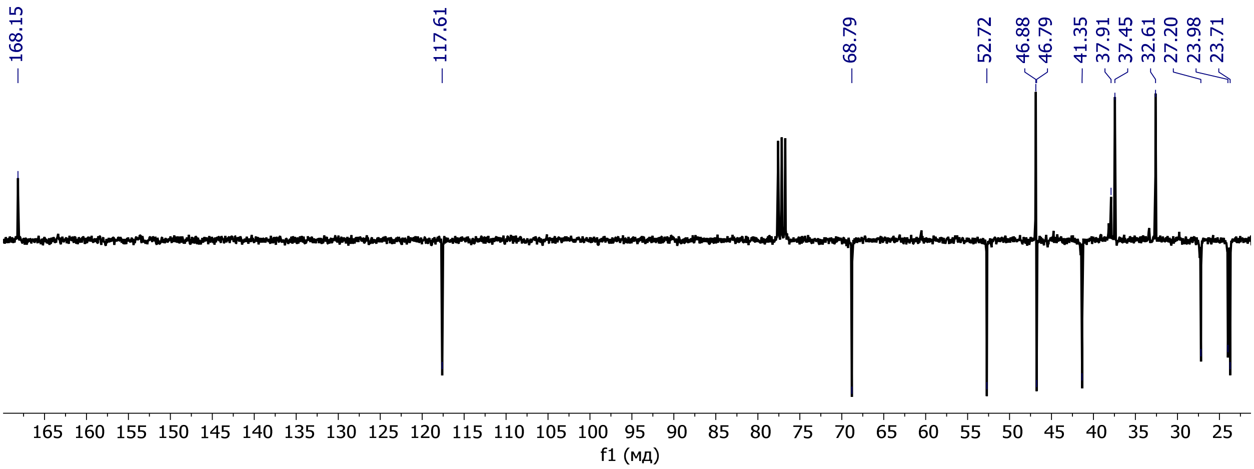
168.15
117.61
68.79
52.72
46.88 46.79
41.35 37.91 37.45 32.61 27.20 23.98 23.71
f1 (мд)

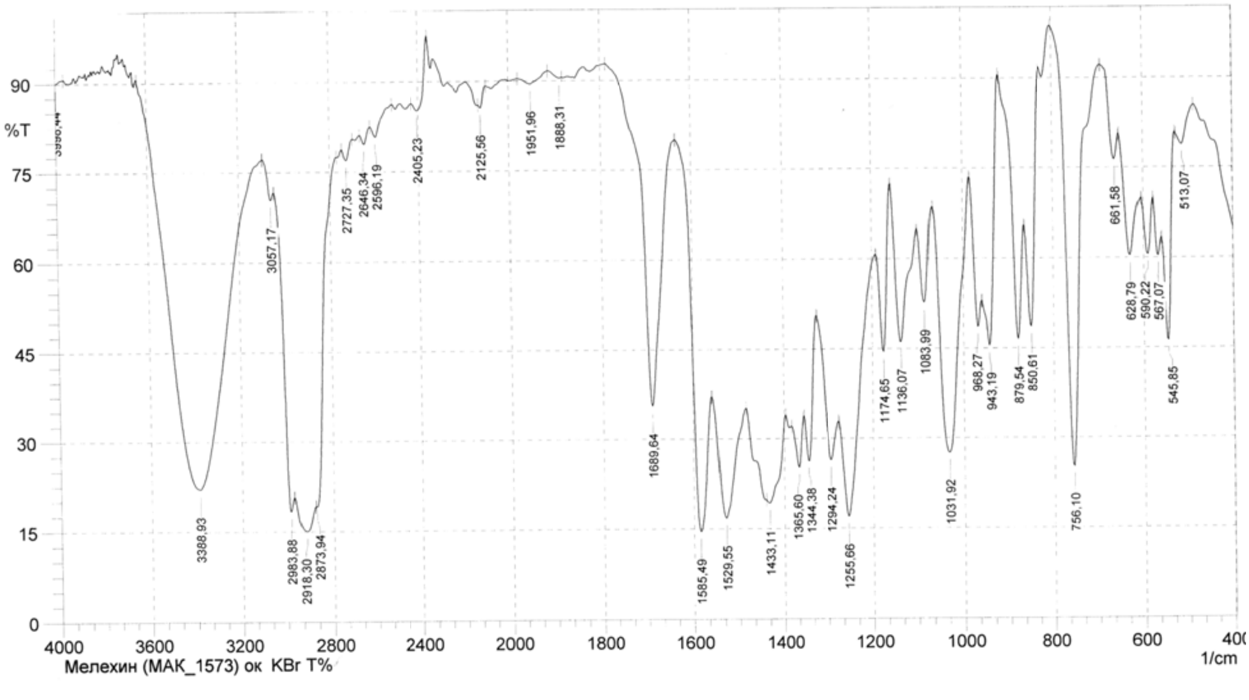
%T
3388,93
3057,17
2983,88
2918,30
2873,94
2727,35
2646,34
2596,19
2405,23
2125,56
1951,96
1888,31
1689,64
1585,49
1529,55
1433,11
1365,60
1344,38
1294,24
1255,66
1174,65
1136,07
1083,99
1031,92
968,27
943,19
879,54
850,61
756,10
661,58
628,79
590,22
567,07
545,85
513,07
1/cm
Мелехин (МАК_1573) ок KBr T%

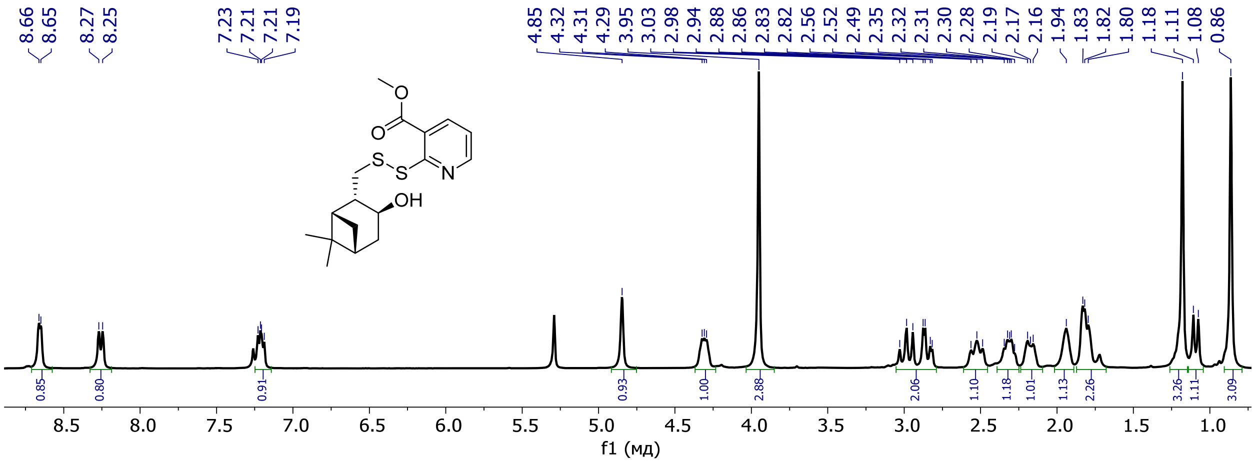
8.66
8.65
8.27
8.25
7.23
7.21
7.21
7.19
4.85
4.32
4.31
4.29
3.95
3.03
2.98
2.94
2.88
2.86
2.83
2.82
2.56
2.52
2.49
2.35
2.32
2.31
2.30
2.28
2.19
2.17
2.16
1.94
1.83
1.82
1.80
1.18
1.11
1.08
0.86
O
O
S
S
N
OH
0.85
0.80
0.91
0.93
1.00
2.88
2.06
1.10
1.18
1.01
1.13
2.26
3.26
1.11
3.09
f1 (мд)

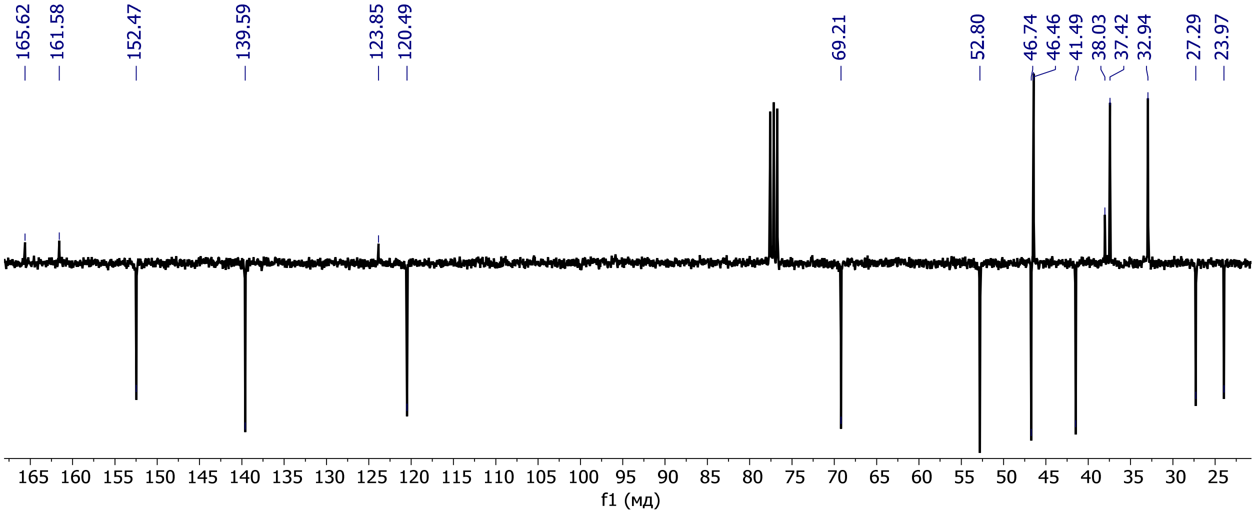
165.62
161.58
152.47
139.59
123.85
120.49
69.21
52.80
46.74
46.46
41.49
38.03
37.42
32.94
27.29
23.97
f1 (мд)

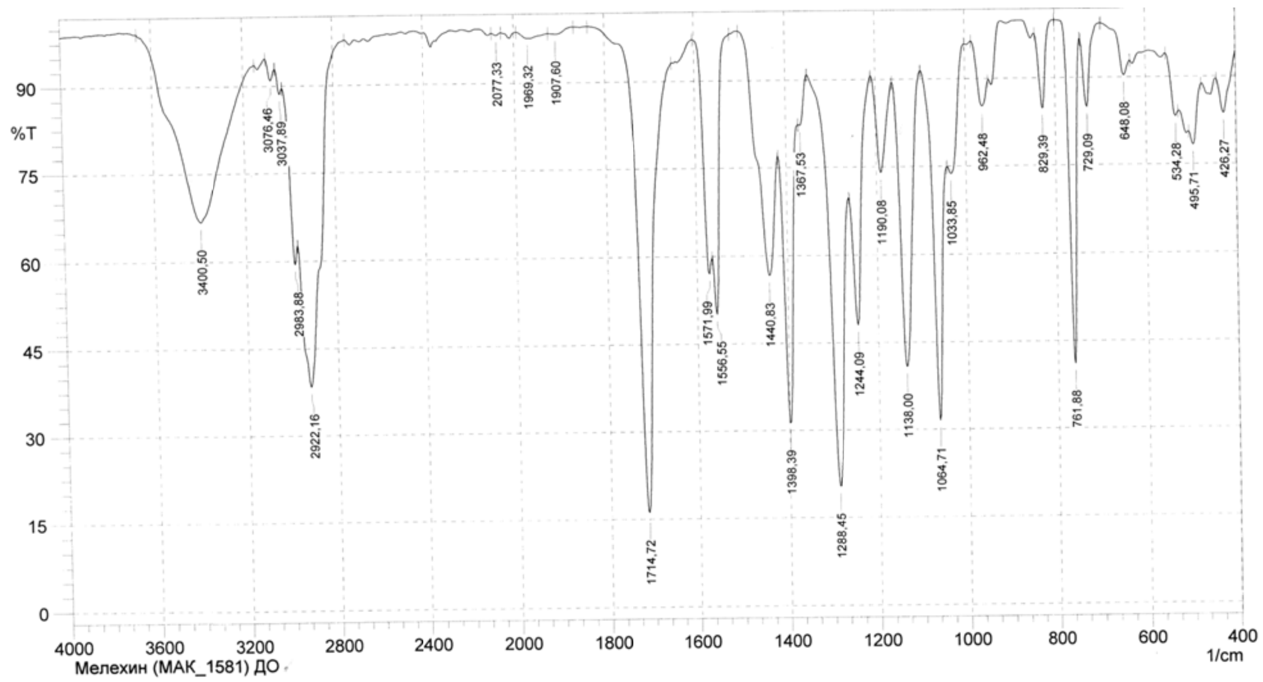
%T
3400,50
3076,46
3037,89
2983,88
2922,16
2077,33
1969,32
1907,60
1714,72
1571,99
1556,55
1440,83
1398,39
1367,53
1288,45
1244,09
1190,08
1138,00
1064,71
1033,85
962,48
829,39
761,88
729,09
648,08
534,28
495,71
426,27
1/cm
Мелехин (МАК_1581) ДО

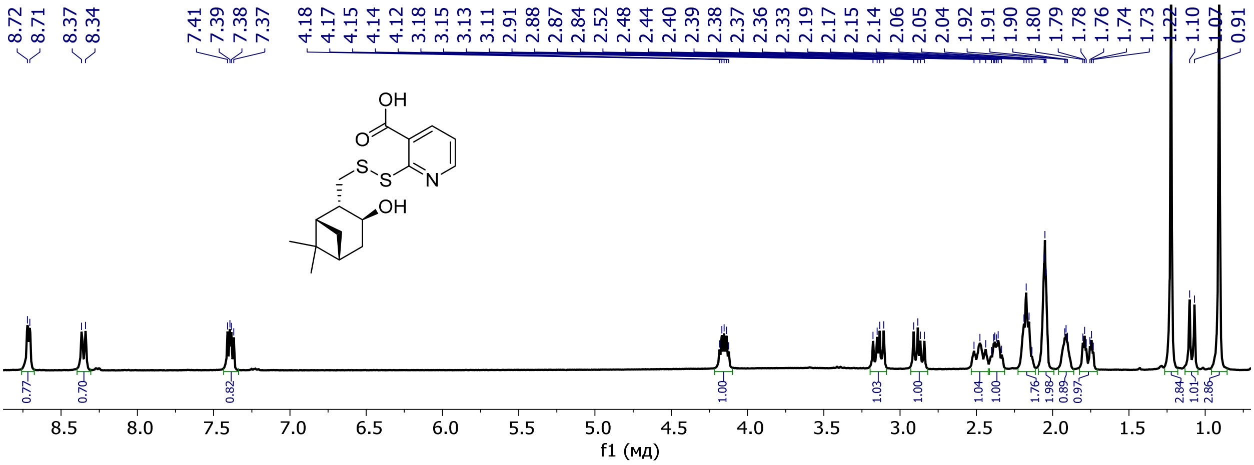
8.72
8.71
8.37
8.34
7.41
7.39
7.38
7.37
4.18
4.17
4.15
4.14
4.12
3.18
3.15
3.13
3.11
2.91
2.88
2.87
2.84
2.52
2.48
2.44
2.40
2.39
2.38
2.37
2.36
2.33
2.19
2.17
2.15
2.14
2.06
2.05
2.04
1.92
1.91
1.90
1.80
1.79
1.78
1.76
1.74
1.73
1.22
1.10
1.07
0.91
OH
O
S
S
N
OH
0.77
0.70
0.82
1.00
1.03
1.00
1.04
1.00
1.76
1.98
0.89
0.97
2.84
1.01
2.86
f1 (мд)

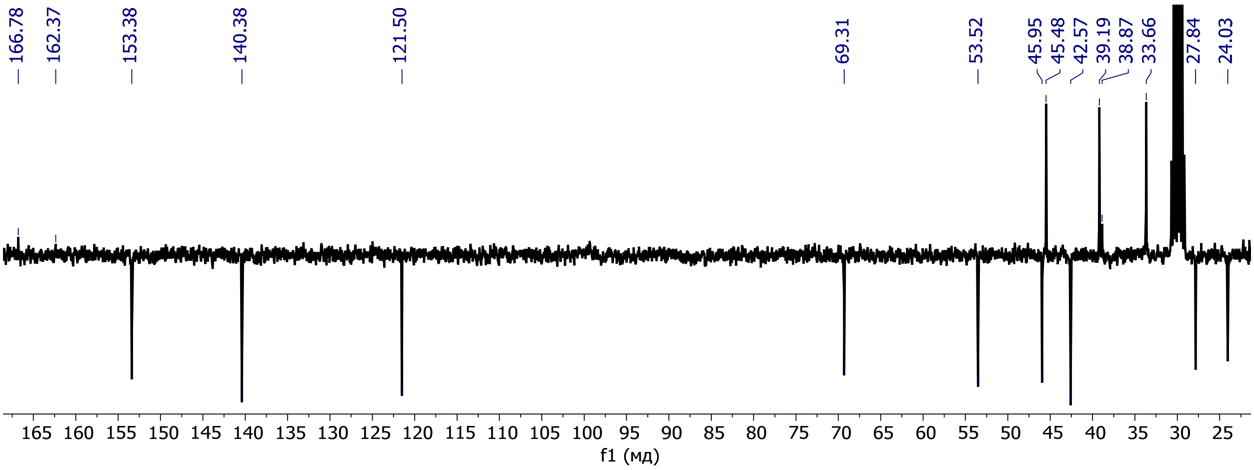
166.78
162.37
153.38
140.38
121.50
69.31
53.52
45.95
45.48
42.57
39.19
38.87
33.66
27.84
24.03
f1 (мд)

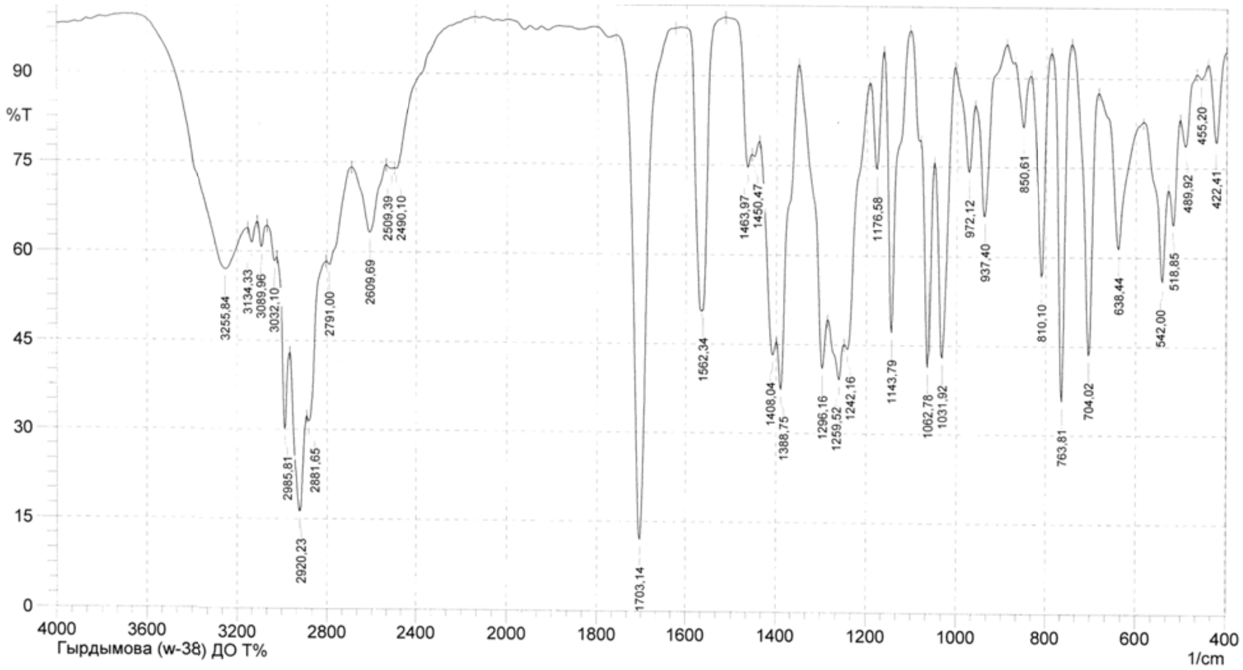
%T
3255,84
3134,33
3089,96
3032,10
2985,81
2920,23
2881,65
2791,00
2609,69
2509,39
2490,10
1703,14
1562,34
1463,97
1450,47
1408,04
1388,75
1296,16
1259,52
1242,16
1176,58
1143,79
1062,78
1031,92
972,12
937,40
850,61
810,10
763,81
704,02
638,44
542,00
518,85
489,92
455,20
422,41
Гырдымова (w-38) ДО T%
1/cm
